# Single Shot vs. Cocktail: A Comparison of Mono- and Combinative Application of miRNA-Targeted Mesyl Oligonucleotides for Efficient Antitumor Therapy

**DOI:** 10.3390/cancers14184396

**Published:** 2022-09-09

**Authors:** Svetlana Gaponova, Olga Patutina, Aleksandra Sen’kova, Ekaterina Burakova, Innokenty Savin, Andrey Markov, Elena Shmendel, Mikhail Maslov, Dmitry Stetsenko, Valentin Vlassov, Marina Zenkova

**Affiliations:** 1 Institute of Chemical Biology and Fundamental Medicine SB RAS, Lavrentiev’s Ave. 8, Novosibirsk 630090, Russia; 2 Department of Physics, Novosibirsk State University, Pirogova Str. 1, Novosibirsk 630090, Russia; 3 Sector of Plant Chemical Biology, Institute of Cytology and Genetics SB RAS, Lavrentiev Ave. 10, Novosibirsk 630090, Russia; 4 Department of Chemistry and Technology of Biologically Active Compounds, Medical and Organic Chemistry Named after N. A. Preobrazhensky, MIREA–Russian Technological University, Vernadsky Ave. 78, Moscow 119454, Russia

**Keywords:** antisense oligonucleotide, mesyl phosphoramidate, oligonucleotide cocktail, anti-miRNA therapy, oncogenic microRNA, lymphosarcoma, melanoma

## Abstract

**Simple Summary:**

Current approaches to the treatment of oncological diseases are still suffering from a lack of efficiency and selectivity and are accompanied by pronounced non-specific toxic effects. This study evaluated the antitumor potential of highly selective multitarget antisense downregulation of small non-coding RNA molecules—microRNAs—where dysregulation in cells frequently triggers oncotransformation and tumor development. We report herein that combinations of recently developed mesyl phosphoramidate oligonucleotides, targeted to multifunctional miRNA regulators miR-17, miR-21 and miR-155, exhibited potent synergistic antiproliferative and antimigrative effects on highly aggressive tumor cells. Furthermore, the significant antitumor activity of a cocktail of three antisense oligonucleotides targeted to miR-21, miR-17, and miR-155 almost completely suppressed lymphosarcoma RLS_40_ tumor growth and exerted prominent antimetastatic effects in a melanoma B16 model. Such treatment elicited no sign of in vivo toxicity and even exhibited remedial effects on the liver of tumor-bearing mice.

**Abstract:**

Rational combinations of sequence-specific inhibitors of pro-oncogenic miRNAs can efficiently interfere with specific tumor survival pathways, offering great promise for targeted therapy of oncological diseases. Herein, we uncovered the potential of multicomponent therapy by double or triple combinations of highly potent mesyl phosphoramidate (µ) antisense oligodeoxynucleotides targeted to three proven pro-oncogenic microRNAs—miR-17, miR-21, and miR-155. A strong synergism in the inhibition of proliferation and migration of B16 melanoma cells was demonstrated in vitro for pairs of µ-oligonucleotides, which resulted in vivo in profound inhibition (up to 85%) of lung metastases development after intravenous injection of µ-oligonucleotide-transfected B16 cells in mice. A clear benefit of µ-21-ON/µ-17-ON and µ-17-ON/µ-155-ON/µ-21-ON combination antitumor therapy was shown for the lymphosarcoma RLS_40_ solid tumor model. In vivo administration of the µ-17-ON/µ-155-ON/µ-21-ON cocktail into RLS_40_-bearing mice elicited fourfold delay of tumor growth as a result of strong inhibition of tumor mitotic activity. It was discovered that the cocktail of µ-21-ON/µ-17-ON/µ-155-ON led to a twofold decrease in total destructive changes in murine liver, which indicates both the reduction in toxic tumor burden and the absence of specific toxicity of the proposed therapy.

## 1. Introduction

Initiation and development of neoplasia is associated with a violation of fundamental cellular processes resulting in enhanced proliferation, apoptosis evasion, immortalization as well as concurrent loss of cell adhesion and an increase in migration potential [1]. Insufficient efficiency and the lack of specificity of existing antitumor programs necessitates the search for novel therapeutics, providing complex targeted influence on carcinogenesis-associated processes. Antisense oligonucleotide technology has proven to be a specific and potent tool for inhibition of tumor-related RNAs, including oncogenic microRNAs (hereafter, miRNAs or miRs) [2,3]. These small non-coding RNAs are actively involved in the regulation of fundamental cellular processes and each miRNA may control the expression of several proteins at a time or be engaged in the regulation of one certain target along with other miRNAs [4]. This is why violation of miRNA functions turns them into the key drivers of various diseases, including cancer. Some particular miRNAs demonstrate functional specificity in cells and, as a consequence, their antisense inhibition is mirrored in the alteration of only one certain process. For instance, application of antisense oligonucleotides against miR-27b resulted solely in the decrease in angiogenesis during lung cancer; treatment with anti-miRNA oligonucleotide (anti-miR-ON), targeted to miR-181b, led to inhibition of metastasis in the case of glioblastoma [5,6]. At the same time, there are miRNAs that represent multifunctional regulators. Antisense oligonucleotide-mediated downregulation of miR-155 in renal cancer cells stimulated apoptosis and inhibited proliferation and migration of tumor cells [7]. Similar treatment of hepatocellular carcinoma cells promoted a decrease in their invasive, proliferative and migration potentials [8]. A number of miRNAs may act as a function-specific player or multi-regulator depending on the type of neoplasia. Inhibition of miR-21 in prostate cancer cells caused only a decrease in their invasive potential. At the same time, application of anti-miR-21-ONs in glioblastoma cells stimulates apoptosis and affects the proliferation and invasiveness of tumor cells [9,10,11]. So, the effects of miRNA-targeted therapy might be quite diverse, but it must be stressed that downregulation of even a single miRNA by anti-miR-ON provides efficient inhibition of pathological processes in tumor cells [12].

In order to enhance the efficiency of miRNA-targeted antitumor therapy, the researchers draw their attention toward the creation of various combinations consisting of miRNA-based (synthetic miRNA mimics) or miRNA-targeted oligonucleotides (anti-miR-ONs) [12]. The following compositions are currently being developed: (1) combinations of an anti-miR-ON or miRNA mimic with a chemotherapeutic agent [13,14]; (2) pairs of synthetic mimics of tumor-suppressor miRNAs [15,16]; and (3) cocktails of several anti-miR-ONs [17,18].

At the moment, the most frequently developed combinations are the cocktails of a single miRNA-based oligonucleotide and a widely used chemotherapeutic agent. Greater emphasis on this type of combination therapy might be explained by the high efficiency of chemotherapy, which remains the gold standard in the treatment of various types of malignant diseases. In such combinations, miRNA-based/targeted oligonucleotides enhance the sensitivity of tumor cells to the applied chemotherapeutic agent via restoration/inhibition of miRNAs, regulating the expression of drug resistance-related genes, whereas chemotherapy promotes a major antitumor effect. Such combinations as miR-205 mimic and gemcitabine, miR-let-7b mimic and paclitaxel, miR-218 mimic and temozolomide, and anti-miR-21-ON and gemcitabine were shown to exert synergetic inhibition of tumor growth and metastasis [13,14,19,20]. However, even if preliminary treatment with oligonucleotides does increase cell sensitivity to chemotherapy, the potent inhibition of carcinogenesis by such combinations is still attained at relatively high doses of chemotherapeutic agents that may exert toxic effects on adjacent healthy tissues.

The less toxic and more specific effect on tumor growth and metastasis can be achieved by applying cocktails combining either tumor-suppressor mimics or anti-miRNA oligonucleotides. The cocktails of two or three miRNA mimics or anti-miR-ONs affect the level of particular miRNA targets, and do not require high doses of compounds for considerable antitumor effect [16,18]. These combinations are quite promising since they may both concurrently regulate several cellular functions managed by different miRNAs or provide reinforced inhibition of one cellular event, if applied oligonucleotides are targeted to members of the same miRNA family [21,22,23].

In this work, we investigated the antitumor potential of mono- and combined therapy with anti-miR-ONs targeted to highly oncogenic miR-17, miR-21 and miR-155, which are potent multifunctional regulators of cellular processes. To inhibit miRNA activity, we applied recently developed chemically modified antisense oligonucleotides, containing mesyl phosphoramidate (µ) modification of internucleotidic phosphates. These antisense µ-ONs were proven to exhibit high therapeutic potential in a number of studies [24,25,26,27]. Notably, our recent research showed the outstanding stability, superior RNase H-activating ability, long-lasting and specific downregulation of miRNA targets, and high biological performance of µ-oligonucleotides in tumor cells. Moreover, the pronounced antitumor effect and low toxicity of this modification were demonstrated in a xenograft model of human epidermoid carcinoma KB-8-5 in vivo using miR-21-targeted µ-oligonucleotide [25].

These prominent results allowed us to expect that combinations of miRNA-targeted µ-oligonucleotides may act synergistically and provide better antitumor effects than monotherapy. Here, for the first time, we compared the efficiency of combined and monotherapy with µ-17-ON, µ-21-ON and µ-155-ON on the murine multidrug-resistant lymphosarcoma RLS_40_ and melanoma B16 tumor models and resolved the dilemma: to mix or not to mix the µ-anti-miR-ONs in order to reach the potent inhibition of malignant properties of cancer cells.

## 2. Materials and Methods

### 2.1. Oligonucleotide Synthesis

μ-oligonucleotides were synthesized by automated solid-phase synthesis according to the protocol published previously [24,25]. The following oligonucleotides were prepared for the study (symbol μ corresponds to the mesyl phosphoramidate internucleotidic groups):

μ-21-ON 5′- TμCμAμAμCμAμTμCμAμGμTμCμTμGμAμTμAμAμGμCμTμA-3′; μ-155-ON 5′- AμAμCμCμCμCμTμAμTμCμAμCμGμAμTμTμAμGμCμAμTμTμAμA-3′; μ-17-ON 5′- CμTμAμCμCμTμGμCμAμCμTμGμTμAμAμGμCμAμCμTμTμTμG-3′; μ-Scr-ON 5′- CμAμAμGμTμCμTμCμGμTμAμTμGμTμAμGμTμGμGμTμT-3′.

### 2.2. Transfection of Tumor Cells with Oligonucleotides

Murine B16 melanoma cells were obtained from the Cell Culture Bank of the Blokhin National Medical Oncology Research Center, Moscow, Russia. Cells were pre-seeded in Dulbecco’s Modified Eagle Medium (DMEM) containing 10% fetal bovine serum (FBS) a day before transfection and were incubated at 37 °C in a humidified atmosphere with 5% CO_2_ (standard conditions). Before the transfection, the medium was replaced with serum-free DMEM. Murine lymphosarcoma RLS_40_ cells were established in the Institute of Chemical Biology and Fundamental Medicine SB RAS, Novosibirsk, Russia [28]. Cells were seeded in serum-free Iscove’s Modified Dulbecco’s Medium (IMDM) immediately before transfection. Transfection was performed by incubation of cells with 10–150 nM µ-oligonucleotides precomplexed with Lipofectamine™2000 (Invitrogen, Waltham, MA, USA) in Opti-MEM medium (Invitrogen, Waltham, MA, USA) according to the manufacturer’s instructions. In the study of the combinative effects of µ-oligonucleotides, the cells were treated with paired combinations, where each of the compounds was taken in concentrations of 25, 50, 75 and 100 nM. The resulting matrix yielded such concentration ratios (in nM) as 25 + 25, 25 + 50, 25 + 75, 50 + 50, 50 + 75, 75 + 75 and 100 + 100. Each oligonucleotide was also applied in a monotherapy regime at the same total concentrations.

For in vitro experiments, 4 h after transfection, the medium was replaced by culture medium containing 10% FBS and 1% antibiotic antimycotic solution (10,000 mg/mL streptomycin, 10,000 IU/mL penicillin, and 25 µg/mL amphotericin), and the cells were cultivated for 96 h under standard conditions. For ex vivo experiments, 4 h after transfection, the medium was removed and cells were diluted in saline buffer for further injections to mice.

### 2.3. xCelligence Real-Time Analysis of Cell Proliferation and Viability

Proliferation experiments were performed using an xCelligence real-time cell analysis (RTCA) system (ACEA Biosciences, Santa Clara, CA, USA) in an atmosphere of 5% CO_2_ at 37 °C. B16 melanoma cells were seeded at a concentration of 5 × 10^3^ cells per well of 16-well E-Plates. The following day, the cells were transfected with oligonucleotides alone or in combination, as described above. Cell proliferation experiments were run for 96 h and cell index was monitored every 30 min for the whole experiment duration. The combined effect of oligonucleotides was assessed by the Bliss and Loewe models analyzed with the SynergyFinder software (https://synergyfinder.fimm.fi/, accessed on 14 February 2022) [29]. The synergy score < −10 denotes antagonism; −10–10—additive effect; and >10—synergy.

### 2.4. Scratch Assay

B16 melanoma cells at a density of 7 × 10^5^ per well were seeded in a serum-free IMDM onto a 6-well plate. When cell confluency reached 80%, cells were transfected with oligonucleotides alone or in combinations as described above. Twenty-four hours after transfection, three wound gaps in each well were scratched vertically with a micropipette tip. The floating cells were washed away by sterile phosphate-buffered saline (PBS) twice before adding fresh serum-free IMDM. At 0 and 24 h after scratch application, the cells were photographed using a phase-contrast microscope (Zeiss Primo Vert, Zeiss, Germany) and analyzed with ImageJ (National Institute of Health, Bethesda, MD, USA). Along the length of each scratch, at least five photographs were taken and the width of each scratch was calculated as a mean value ± standard error of five meanings obtained for one time point. For each well, the average scratch width for the given time point was measured as mean value ± standard error of three independent scratches performed in each well. The migration area was estimated as the ratio of the area filled with cells after 24 h to the initial scratch area. The migration rate of the cells was estimated as the degree of wound healing and calculated according to the formula: υ = (1 − Χ) × 100%, where Χ is the ratio of the scratch width at 24 h to the scratch width at 0 h. The interaction of oligonucleotides in combination was evaluated by the Loewe model, analyzed with the SynergyFinder software (https://synergyfinder.fimm.fi/, accessed on 14 February 2022) [29]. The synergy score < −10 denotes antagonism; −10–10—additive effect; and >10—synergy.

### 2.5. Mice

Female 10–12-week-old CBA/LacSto (hereinafter, CBA) and C57Bl/6 mice were kept in the vivarium of the Institute of Chemical Biology and Fundamental Medicine, SB RAS, with a natural light regime on a standard diet for laboratory animals (GOST (State Standard) R 5025892) in compliance with the international recommendations of the European Convention for the Protection of vertebrate animals used for experimental studies (1997), as well as the rules of laboratory practice in the performance of preclinical studies in the Russian State Standards (R 51000.3–96 and 51000.4–96). The experimental protocols #124 and #125 were approved by the Committee on the Ethics of Animal Experiments with the Institute of Cytology and Genetics of the Siberian Branch of the Russian Academy of Sciences.

### 2.6. Antitumor Studies Ex Vivo

To investigate the effect of oligonucleotides on primary tumor growth and lung metastases formation, the RLS_40_ and B16 cells, respectively, were used. RLS_40_ ascites were taken from CBA mice intraperitoneally injected with tumor cells (2 × 10^6^ in 0.2 mL saline buffer) into abdominal cavities. RLS_40_ cells, isolated from ascites fluid by filtration through Lymphocyte Separation Medium (LSM) (MP Biomedicals, Irvine, CA, USA), as well as B16 cells, were divided into ten parts: (1) untreated cells (Control); (2) the cells incubated with Lipofectamine™2000; (3)–(6) the cells transfected with µ-Scr-ON, µ-21-ON, µ-17-ON, and µ-155-ON in the 150 nM concentration; (7)–(9) the cells transfected with double cocktails μ-21-ON/μ-17-ON, μ-21-ON/μ-155-ON, and μ-17-ON/μ-155-ON in the 75 nM concentration of each compound; and (10) the cells transfected with triple combination μ-21-ON/μ-17-ON/μ-155-ON in a 50 nM concentration of each compound. After 4 h of transfection, B16 cell suspensions of 2 × 10^5^ cells (0.1 mL) were intravenously administered to C57Bl/6 mice for lung metastasis formation, and RLS_40_ cell suspensions of 10^6^ cells (0.1 mL) were intramuscularly inoculated into the right thigh of CBA mice for solid tumor development. As soon as tumors began to be palpable, the tumor volumes were measured every 2–3 days using calipers. Tumor volumes were calculated as V = π/6 × length × width × height.

At day 20, the mice were euthanized and tumors and internal organs, including lungs, were collected and fixed in 10% neutral-buffered formalin (BioVitrum, St. Petersbur, Russia) for further histological analysis. Lungs of B16 melanoma-bearing mice were collected at the end of the experiment for calculation of surface metastases using a binocular microscope.

A part of transfected B16 and RLS_40_ cells were cultivated for 72 h to further extraction of total RNA and measurement of miRNA level by stem-loop PCR, as described in [25,30].

### 2.7. Preparation of the Complexes of Cationic Liposomes and Oligonucleotides

Prior to use in in vivo studies, the cationic folate-equipped liposomes F [31] and µ-oligonucleotides were mixed to form lipoplexes in a serum-free Opti-MEM by mixing equal volumes of liposomes and respective oligonucleotide solutions. Lipoplexes were prepared at an N/P ratio of 4/1, where N/P is a ratio between nitrogen atoms of polycationic and helper lipids in liposomes and phosphorus atoms of oligonucleotides. The resulting mixtures were incubated for 20 min at room temperature and used for in vivo treatment.

### 2.8. Antitumor Studies In Vivo

The antitumor effect of µ-oligonucleotides in vivo was evaluated using lymphosarcoma RLS_40_. Tumors were initiated in CBA mice by intramuscular injection of RLS_40_ cells, 10^6^ in 0.1 mL in sterile saline buffer, into the right thigh. At day 4 after transplantation, the mice were divided into four groups according to further treatment (n = 10 mice per group): (1) mice injected with folate-equipped liposomes F (Control); (2) and (3) mice injected with µ-Scr-ON and µ-21-ON at a dose of 10 µg per mouse; and (4) mice injected with triple combination μ-21-ON/μ-17-ON/μ-155-ON at a dose of 3.3 µg per mouse of each compound. Oligonucleotides were precomplexed with liposomes F as described above at an N/P ratio of 4/1. In total, three peritumoral injections of oligonucleotides were made with a 3-day interval. The tumor volumes were measured every 3 days using calipers in an investigator-blinded fashion. Tumor volumes were calculated as V = π/6 × length × width × height.

At day 24, animals were euthanized and tumors were collected and fixed in 10% neutral-buffered formalin for further histological analysis.

### 2.9. Histology, Immunohistochemistry and Morphometry

For histological analysis, the tumor, lung, liver and kidney specimens were collected and fixed in 10% neutral-buffered formalin, dehydrated in ascending ethanols and xylols and embedded in HISTOMIX paraffin (BioVitrum, St. Petersbur, Russia). Paraffin sections (5 μm) were sliced using a Microm HM 355S microtome (Thermo Fisher Scientific, Waltham, MA, USA) and stained with hematoxylin and eosin. For immunohistochemical (IHC) study, tumor sections (3–4 μm) were deparaffinized and rehydrated; antigen retrieval was carried out after exposure in a microwave oven at 700 W. The samples were incubated with anti-PCNA-specific antibodies (ab92552, Abcam, Cambridge, UK) according to the manufacturer’s protocol. Next, the sections were incubated with secondary horseradish peroxidase (HPR)-conjugated antibodies, exposed to the 3,3′-diaminobenzidine (DAB) substrate (Rabbit Specific HRP/DAB (ABC) Detection IHC Kit, ab64261, Abcam, Cambridge, UK), and stained with Mayer’s hematoxylin. All the images were examined and scanned using Axiostar Plus microscope equipped with Axiocam MRc5 digital camera (Zeiss, Germany) at magnification ×400.

Inhibition of metastases development of B16 melanoma in lungs of mice was assessed by morphometric analysis using the metastasis inhibition index (MII), calculated as MII = ((mean metastasis area control–mean metastasis area experiment)/mean metastasis area control) × 100%. The MII of the µ-Scr-treated group was taken as 0% and the MII that reflected the absence of metastases was taken as 100%.

Morphometric analysis of tumor and liver sections was performed using a counting grid consisting of 100 testing points in a testing area equal to 3.2 × 10^6^ μm^2^. A total of 5–10 random fields were studied in each sample depending on sample size, forming 50–100 random fields for each group of mice in total. Morphometric analysis of tumor tissue included evaluation of the numerical density (Nv) of mitoses and the number of PCNA-positive cells with subsequent calculation of proliferation index according to the formula: number of PCNA-positive cells/total number of cells × 100%. Morphometric analysis of liver tissue included evaluation of the volume densities (Vv, %) of normal hepatocytes, dystrophy and necrosis in the liver parenchyma, as well as the numerical densities (Nv) of binuclear hepatocytes reflecting the regeneration capacity of the liver. The volume density (Vv, %) of studied histological structures representing the volume fraction of tissue occupied by this compartment was calculated using the formula: Vv = (Pstructure/Ptest) × 100%, where Pstructure denotes the number of testing points over the structure and Ptest denotes the total number of testing points—in this case, 100. The numerical density (Nv) of studied histological structures indicating the number of particles in the volume unit of the tissue was evaluated as a number of particles in the square unit, 3.2 × 10^6^ μm^2^ in this case.

### 2.10. Reconstruction of miRNA-Target Networks and Their Functional Annotation

Experimentally verified target genes of µ-anti-miR-ONs-susceptible miRNAs were retrieved from the miRTarBase v. 8.0 database [32] (*Mus musculus*) using the CyTargetLinker v. 4.1.0 plugin [33] and visualized with Cytoscape v. 3.7.2. Functional analysis of revealed target genes was performed using the ClueGO v. 2.5.7 plugin [34] based on four independent databases, including the Gene Ontology (Biological Processes), Kyoto Encyclopedia of Genes and Genomes (KEGG), REACTOME, and WikiPathways. The significance of gene-term enrichment was evaluated with a two-sided hypergeometric test corrected by the Bonferroni method. Only functional terms with *p* ≤ 0.05 were collected and grouped into clusters using kappa statistics (κ score = 0.4). Finally, the interactions between retrieved target genes were restored using the STRING database [35] and the reconstructed regulome was analyzed using the NetworkAnalyzer tool [36] in Cytoscape.

### 2.11. qPCR

After in vitro transfection of B16 and RLS_40_ cells as described in Section 2.2, total RNA was extracted from tumor cells at time points of 24 and 72 h using TRIzol Reagent (Invitrogen, Waltham, MA, USA) according to the manufacturer’s protocol. The level of miRNAs was measured using stem-loop qPCR technology [37,38]. cDNA synthesis was carried out using MuML-V reverse transcriptase (Biolabmix, Novosibirsk, Russia) according to the manufacturer’s protocol. The RT and PCR primers used in the study are listed in Table S1. PCR amplification was carried out using BioMaster HS-qPCR SYBR Blue mix (Biolabmix, Novosibirsk, Russia) according to the manufacturer’s protocol. The obtained qPCR data were analyzed by standard Bio-Rad iQ5 v.2.0 software. For each sample, the threshold cycle (Ct) was determined. Quantitative assessment of the level of transcript representation, relative miRNA and mRNA expression was performed by comparing the Ct values for miRNA and mRNA with *HPRT1* and *GAPDH* mRNAs used as references.

### 2.12. Western Blot

Cell lysates from RLS_40_ and B16 cells were collected in radioimmunoprecipitation assay (RIPA) buffer (Thermo Scientific, Waltham, MA, USA), separated in 12.5% sodium dodecyl sulfate (SDS)/PAGE and transferred to a poly(vinylidene difluoride) (PVDF) membrane using a semidry transfer. Western Blot analysis was performed as described earlier [30,39] using primary antibodies against PTEN (ab154812, 1:800; Abcam, Cambridge, UK), Stat3 (ab68153, 1:500; Abcam, Cambridge, UK), and GAPDH (ab9485, 1:1000; Abcam, Cambridge, UK), and secondary HRP-conjugated goat anti-rabbit antibodies (ab6721; Abcam, Cambridge, UK).

### 2.13. Protein Level Measurement Using Flow Cytometry

B16 and RLS_40_ cells were transfected as described in Section 2.2, incubated for 72 h and fixed in 4% formaldehyde. Collected samples were incubated in permeabilization buffer (PBS, 2% bovine serum albumin (BSA), 0.1% Tween-20) for 15 min at room temperature and in block buffer (PBS, 4% BSA, 0.05% Tween-20) for 30 min at room temperature. MMP9 protein level was measured by flow cytometry analysis (“Novocyte” flow cytometer (ACEA Biosciences Inc, San Diego, CA, USA)) using primary antibodies (ab228402, 1:200, Abcam Cambridge, UK) and secondary AF488-goat anti-IgG antibodies (ab150077, Abcam Cambridge, UK).

### 2.14. Statistics

The data were statistically analyzed using one-way ANOVA with a post hoc Tukey test. *p* < 0.05 was considered to be statistically significant. The analysis was carried out with application of the STATISTICA v.12.0 software.

## 3. Results

### 3.1. Conceptual Design of the Study

The principal objective of the study was to evaluate the consequences of mono and combined therapy of tumors of different histogenesis with anti-miR-ONs, targeted to oncogenic miR-17, miR-21, and miR-155. Chosen miRNAs represent potent tuners of cellular functions that manage a wide spectrum of protein targets and signaling pathways. Notably, miR-17 mostly influences the p21WAF1/CIP1-dependent, TIMP2/MMPs, and STAT3 pathways, related to cellular proliferation, migration and invasion [40,41]. MiR-21 is most often responsible for the regulation of the processes mediated by MARKS-associated and PTEN/PI3K/Akt pathways, including apoptosis and invasion [9,10,11], whereas miR-155 controls the performance of the VHL/HIF pathway, mostly connected with angiogenesis [42]. Previous studies repeatedly confirmed the high prospects of the downregulation of each of these miRNAs for effective inhibition of processes, associated with tumorigenesis. It was noted that application of a single antisense oligonucleotide against miR-17, miR-21, or miR-155 in different cancer cell lines exhibited antiproliferative, antimigrative, anti-angiogenic and pro-apoptotic effects, which might be realized through the influence on one or several pathways at a time [43,44,45]. The diversity of outcomes of cell treatment with anti-miRNA-ONs used alone makes the consequences of concurrent downregulation of miR-17, miR-21 and miR-155 in cancer cells hard to foresee. Until today, there was no mention of any paired combinations of anti-miR-17-ON, anti-miR-21-ON and anti-miR-155-ON. However, the study of Chaudhary and colleagues illuminated the application of a multitargeted sponge combining the binding sites of all mentioned miRNAs into one structure [13]. It was shown that transfection with this anti-miR-17/miR-21/miR-155 multitargeted sponge was beneficial for efficient inhibition of breast cancer cell proliferation in comparison with monotherapy, with corresponding singular sponges or anti-miRNA-ONs [13].

**Figure 1:**
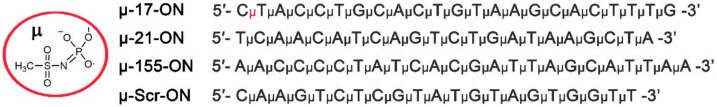
Structure of mesyl phosphoramidate (µ-) modification and sequences of oligonucleotides used in the study. µ-17-ON, µ-21-ON and µ-155-ON are µ-modified oligonucleotides, targeted to miR-17, miR-21 and miR-155, respectively. µ-Scr-ON represents control µ-oligonucleotide that has no targets in the mammalian genome.

In the present work, 22–23-mer linear oligonucleotides (ONs), fully complementary to miR-17, miR-21 and miR-155, containing mesyl phosphoramidate (µ-) modification of all internucleotidic phosphates (µ-17-ON, µ-21-ON and µ-155-ON) were used (Figure 1). The µ-ONs were applied for in vitro or in vivo treatment of tumor models in a monotherapy regimen or in cocktails, containing two or three oligonucleotides. Treatment efficiency was evaluated with regard to 22-mer scramble µ-oligonucleotide (µ-Scr-ON) that has no targets in mammalian genome.

It should be mentioned that murine lymphosarcoma RLS_40_ and melanoma B16 tumor models are characterized by high expression of all three selected miRNAs (miR-17, miR-21 and miR-155) (Figure S1) and were used for the first time to evaluate which strategy is the most efficient to suppress oncogenic processes: silencing of one particular miRNA (miR-17, miR-21, or miR-155) or several of these miRNAs at a time. The study included figuring out if given miRNAs serve as function-specific or multidirectional regulators in RLS_40_ and B16 cells and evaluating the possible interactions of anti-miRNA oligonucleotides in combinations used.

### 3.2. Antiproliferative Effect of Mono- and Combination Therapy with µ-Oligonucleotides Targeted to miR-21, miR-17 and miR-155

The antiproliferative and antimigration effects of anti-miRNA oligonucleotides were studied in vitro using B16 murine melanoma cells, which are characterized by high proliferative and migration indexes and represent the prototype of highly aggressive human melanoma. The proliferative response of B16 cells to treatment was monitored in real time using the xCelligence system. It was revealed that all three oligonucleotides—µ-17-ON, µ-21-ON and µ-155-ON—significantly reduce cell growth rate in a dose-dependent manner. Dose–response curves of cell proliferation inhibition obtained for each oligonucleotide in a dose range 0–150 nM are shown in Figure S2. µ-21-ON exerted the highest antiproliferative effect, while µ-155-ON had the smallest effect on cell growth: the maximum observed proliferation inhibition reached 80, 67 and 47% and IC50 values were 60, 93 and >150 nM for µ-21-ON, µ-17-ON and µ-155-ON, respectively (Figure S2).

**Figure 2:**
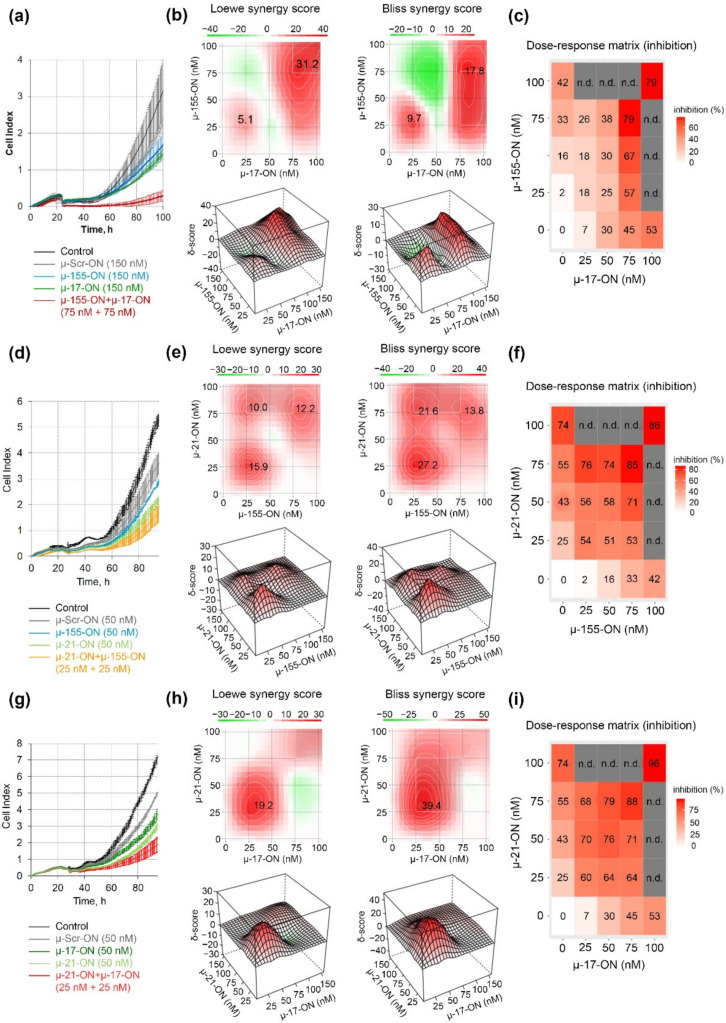
Antiproliferative effect of oligonucleotide cocktails µ-17-ON/µ-155-ON (**a**–**c**), µ-21-ON/µ-155-ON (**d**–**f**) and µ-21-ON/µ-17-ON (**g**–**i**) in B16 melanoma cells. (**a**,**d**,**g**) Representative graphs of proliferation activity of B16 cells recorded in real time using the xCELLigence cell analysis system after treatment with µ-155-ON (150 nM), µ-17-ON (150 nM) and µ-17-ON/µ-155-ON (75 + 75 nM) (**a**); with µ-21-ON (50 nM), µ 155-ON (50 nM) and µ-21-ON/µ-155-ON (25 + 25 nM) (**d**); and with µ-21-ON (50 nM), µ-17-ON (50 nM) and µ-21-ON/µ-17-ON (25 + 25 nM) (**g**). As a control, µ-Scr-ON was used. Control—B16 cells without treatment. Data represent mean ± s.d. (**b**,**e**,**h**) Loewe and Bliss score showing synergistic effect of oligonucleotide combinations µ-17-ON/µ-155-ON, µ-21-ON/µ-155-ON and µ-21-ON/µ-17-ON on cell growth. The heatmap shows the score values for the areas with the highest synergy. The data were analyzed with the SynergyFinder software (https://synergyfinder.fimm.fi/, accessed on 14 February 2022). The synergy score < −10—antagonism; −10–10—additive effect; >10—synergy; (**c**,**f**,**i**) Matrices demonstrating inhibition of cell growth by different concentrations of oligonucleotides in combinations. n.d.—not determined.

The antiproliferative effect was further studied for pairs of the oligonucleotides in a dose range of 25–100 nM, and dose–response matrices were designed for each pair (Figure 2). It turned out that the cytostatic effect of different oligonucleotide pairs displayed a different nature of interactions. Loewe and Bliss model analysis of synergy/antagonism of cell growth inhibition revealed that the combination of µ-17-ON and µ-155-ON demonstrates an additive effect at a 25 nM concentration of each of the oligonucleotides (Figure 2a–c). For this pair, concentrations over 75 nM are required to provide a synergistic effect, characterized by 31.2 and 17.8 Loewe and Bliss synergy score (Figure 2a,c). At these doses, the efficient 80% inhibition of B16 cell proliferation is observed (Figure 2a). Based on the heatmap data and dose–response matrix, it can be assumed that µ-17-ON provides the main effect, and the addition of µ-155-ON significantly increases this effect, promoting synergism (Figure 2b,c).

Both cocktails µ-21-ON/µ-155-ON and µ-21-ON/µ-miR-17-ON demonstrated synergistic co-operative inhibition of B16 cell growth in the whole 0–75 nM concentration range (Figure 2e,f,h,i). For both pairs, the strong synergism is observed already at 25 nM concentration of each oligonucleotide in the combination, when suppression of cellular growth reaches 54% for µ-21-ON/µ-155-ON and 60% for µ-21-ON/µ-miR-17-ON, increasing from 25% for µ-21-ON, 7% for µ-17-ON and 2% for µ-155-ON (Figure 2d,e,g,h). Loewe and Bliss synergy scores in this concentration range are 15.9 and 27.2 for µ-21-ON/µ-155-ON (Figure 2e) and 19.2 and 39.4 for µ-21-ON/µ-miR-17-ON (Figure 2h). At higher concentrations, the synergy score decreases, but still remains as high as 13 for both combinations. Dose–response matrices show that there is a mutual enhancement in antiproliferative effect of oligonucleotides in the studied pairs (Figure 2f,i).

### 3.3. Antimigrative Effect of Mono- and Combination Therapy with µ-Oligonucleotides Targeted to miR-21, miR-17 and miR-155

**Figure 3:**
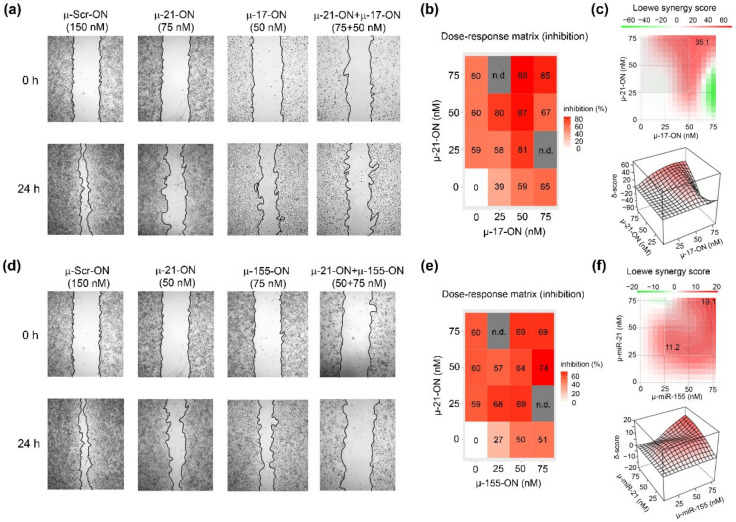
Antimigrative effect of oligonucleotide cocktails µ-21-ON/µ-17-ON and µ-21-ON/µ-155-ON on B16 melanoma cells. (**a**,**d**) Migration activity of B16 cells, determined by wound healing assay, 24 h after Lipofectamine™2000-mediated transfection with µ-21-ON, µ-17-ON and µ-155-ON used separately and in combinations of µ-21-ON/µ-17-ON and µ-21-ON/µ-155-ON. µ-Scr-ON–B16 cells treated with control oligonucleotide µ-Scr-ON (150 nM). Concentrations of µ-oligonucleotides are shown at the top. (**b**,**e**) Matrices demonstrating inhibition of cell migration by different concentrations of oligonucleotides in combinations of µ-21-ON/µ-17-ON and µ-21-ON/µ-155-ON. n.d.—not determined; (**c**,**f**) Loewe score showing synergistic/antagonistic effect of µ-21-ON/µ-17-ON and µ-21-ON/µ-155-ON pairs on cell migration. The heatmap shows the score values for the areas with the highest synergy. The data were analyzed using the SynergyFinder web application. The data were analyzed with the SynergyFinder software (https://synergyfinder.fimm.fi/, accessed on 14 February 2022). The synergy score < −10—antagonism; −10–10—additive effect; >10—synergy.

The antimigrative potential of µ-oligonucleotides was studied first for anti-miRNA-ONs used separately. It was found that all three oligonucleotides provided a significant decrease in melanoma B16 cells’ migration 24 h post-transfection (Figure 3 and Figure S3). The effects of mono-application of µ-17-ON and µ-155-ON were dose-dependent and amounted to 20% at the lowest concentrations (25–35 nM) and reached up to 90% at 150 nM (Figure S3a,b). In turn, the wound healing curve for µ-21-ON reached a plateau at 25 nM concentration, resulting in 60% suppression of migrative activity, persisting up to 135 nM (Figure S3a,b); a further increase in µ-21-ON concentration to 150 nM led to 85% inhibition of wound healing (Figure S3a,b).

Considering the combinative application of µ-oligonucleotides, we observed that µ-17-ON and µ-155-ON played antagonistically, decreasing the antimigrative effect of single oligonucleotides (Figure S4). As an example, the reduction in the migration potential of cells by a combination of oligonucleotides µ-17-ON/µ-155-ON (50 + 75 nM) was 38%, decreasing from 59% for µ-17-ON (50 nM) and 51% for µ-155-ON (75 nM), respectively (Figure S4a,b). On the contrary, cocktails µ-21-ON/µ-17-ON and µ-21-ON/µ-155-ON considerably enhanced the antimigrative effect of treatment (Figure 3). Figure 3a shows that cell transfection with µ-17-ON/µ-21-ON significantly impedes filling of the scratch with B16 cells in contrast to application of each oligonucleotide separately. According to dose–response matrices, the antimigrative effects of cocktails were considerably higher in comparison with singular oligonucleotides and enhanced from 39% for µ-17-ON (25 nM) and 60% for µ-21-ON (50 nM) up to 80% for µ-17-ON/µ-21-ON (25 + 50 nM) and from 27% for µ-155-ON (25 nM) and 59% for µ-21-ON (25 nM) up to 68% for the µ-155-ON/µ-21-ON (25 + 25 nM) cocktail (Figure 3a,c,d,f). Both combinations provide strong inhibition of the wound healing rate of B16 cells, with Loewe synergy score being equal to 35.1 and 19.1 for µ-21-ON/µ-17-ON and µ-21-ON/µ-155-ON, respectively (Figure 3c,f).

### 3.4. Antitumor and Antimetastatic Activity of µ-Oligonucleotides in Murine Models

To further evaluate the antimetastatic and antitumor potential of mono- and combined therapy with µ-oligonucleotides in vivo, two murine tumor models were used: metastatic model of melanoma B16 and solid tumor model of multidrug-resistant lymphosarcoma RLS_40_.

**Figure 4:**
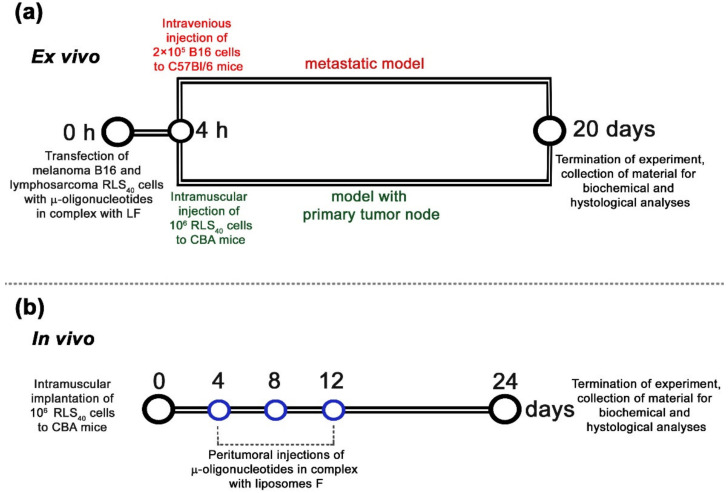
Design of ex vivo (**a**) and in vivo (**b**) experiments. (**a**) Ex vivo experiments included in vitro transfection of tumor cells with oligonucleotides alone or in combinations in complex with Lipofectamine™2000 (LF) at a total concentration of 150 nM followed by intravenous injections of 2 × 10^5^ cells (per mice) to C57Bl/6 mice (melanoma B16, metastatic model) or intramuscular implantation of 10^6^ cells (per mice) to CBA mice (lymphosarcoma RLS_40_, solid tumor model). (**b**) In vivo experiments included engraftment of CBA mice with lymphosarcoma RLS_40_ followed by peritumoral injections of either μ-21-ON or a combination of μ-21-ON/μ-17-ON/μ-155-ON or control μ-Scr-ON in complex with folate-containing liposomes F at a total dose of 10 μg per mouse. In total, three injections were made at days 4, 8, and 12 after tumor implantation.

To study the oncosuppressive activity of oligonucleotides, ex vivo models were used that enabled the elimination of the issue of in vivo targeted delivery of oligonucleotides and revealed the net antitumor effect. This included Lipofectamine™2000-mediated transfection of B16 or RLS_40_ cells with µ-oligonucleotides applied separately or in cocktails followed by intravenous injection of transfected cells to C57Bl/6 mice to initiate melanoma B16 lung metastases or intramuscular inoculation of transfected RLS_40_ cells to CBA mice to form lymphosarcoma RLS_40_ solid tumors (Figure 4a) (hereafter, ex vivo experiment).

In these experiments, each µ-oligonucleotide in pairs was used at a concentration of 75 nM, since, according to in vitro data, the maximum synergistic antiproliferative and antimigrative effect, reaching 80–90%, for µ-21-ON/µ-17-ON, µ-17-ON/µ-155-ON and µ-21-ON/µ-155-ON pairs was observed at this concentration (Figure 2 and Figure 3). In these experiments, a triple combination of µ-21-ON/µ-17-ON/µ-155-ON, where each oligonucleotide was used at a concentration 50 nM, was studied as well. The combined treatment with oligonucleotide cocktails was compared with the effects of µ-oligonucleotide used separately with a total concentration of µ-ONs of 150 nM. The efficiency and specificity of miR-21, miR-17 and miR-155 downregulation in tumor cells after transfection with µ-ONs were verified by stem-loop PCR, as described previously [25,30] (Figures S5 and S6).

### 3.5. The Effect of Anti-miRNA Mono- and Combination Therapy with µ-Oligonucleotides on Melanoma B16 Metastases Development in Mice (Ex Vivo Experiment)

**Figure 5:**
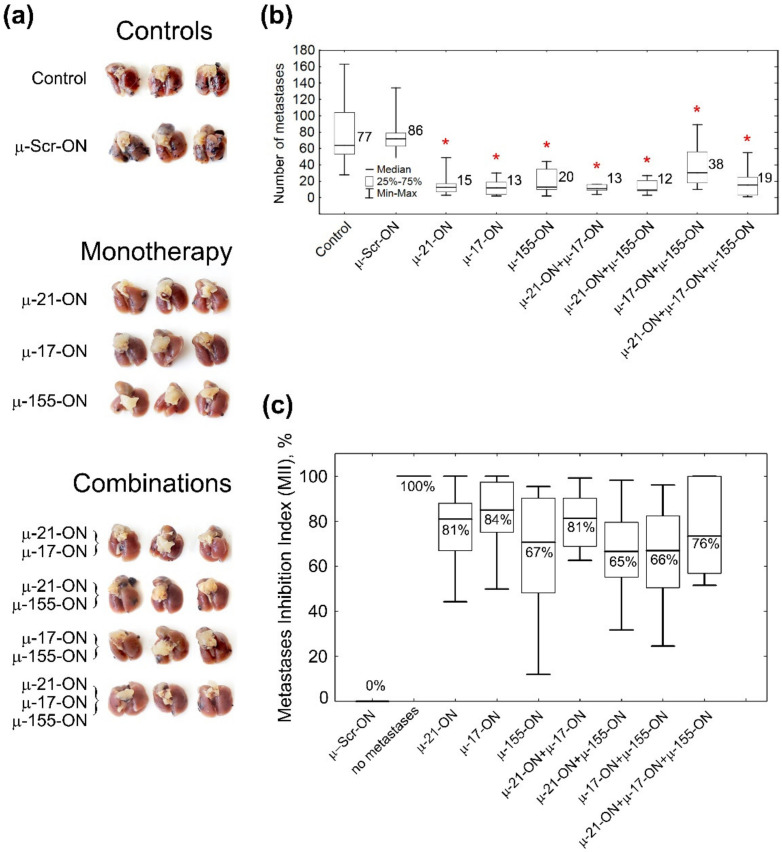
Antimetastatic effect of miRNA-targeted µ-oligonucleotides. (**a**) Photographs of formalin-fixed lungs with melanoma B16 metastases. (**b**) Box plot showing the number of surface metastases in lungs in each group. Numbers on the right side of the boxes show average number of lung surface metastases in the group. Red asterisk shows statistically significant difference from μ-Scr-ON and Control groups with *p* ≤ 0.0001. (**c**) Box plot showing the Metastases Inhibition Indexes (MIIs) in each group. 0% corresponds to the area of metastases in the µ-Scr-ON group, 100% corresponds to the complete absence of metastases. Melanoma B16 cells were transfected with µ-oligonucleotides followed by intravenous injection to C57Bl/6 mice. Control—cells treated with Opti-MEM. Total concentration of µ-oligonucleotides upon transfection was 150 nM: single (µ-Scr-ON, µ-21-ON, µ-17-ON or µ-155-ON) 150 nM; pairs (µ-21-ON/µ-17-ON, µ-21-ON/µ-155-ON, µ-17-ON/µ-155-ON)—75 nM; triple cocktail (µ-21-ON/µ-17-ON/µ-155-ON)—50 nM each. Transfection was performed in complex with Lipofectamine™2000. Numbers below the median in the boxes show average MII in the group. The data were statistically analyzed with one-way ANOVA using a Tukey test.

The antimetastatic activity of µ-oligonucleotides was evaluated by counting the number of lung surface metastases and definition of the area of internal metastases in lung tissue, formed by B16 cells (Figure 4a, Figure 5 and Figure S7). Analysis showed that even a single treatment of B16 cells with µ-oligonucleotides in vitro provided significant inhibition of the metastatic potential of transfected cells in mice. Ex vivo cell transfection with μ-155-ON, μ-21-ON or μ-17-ON (150 nM) promoted 4.3-, 5.7- and 6.6-fold decreases in the number of lung surface metastases in vivo, respectively, compared to control oligonucleotides (13–20 metastases versus 86 metastases in μ-Scr-ON group) (Figure 5a,b and Figure S7). Among oligonucleotide pairs, the lowest inhibitory potential was found for pair μ-155-ON/μ-17-ON, which caused only a two-fold reduction in lung surface metastases. More potently, a 4.5-fold decline in the number of surface metastases was observed for the cocktail μ-17-ON/μ-155-ON/μ-21-ON (Figure 5a,b and Figure S7). The highest efficiency was detected for pairs μ-21-ON/μ-17-ON and μ-21-ON/μ-155-ON, which provided 6.6- and 7.2-fold diminishment in metastatic potential of B16 cells in mice (12–13 metastases versus 86 metastases in the μ-Scr-ON group) (Figure 5a,b and Figure S7).

The Metastases Inhibition Indexes (MIIs) were evaluated using morphometric analysis of the area occupied by internal lung metastases in each group, treated with µ-oligonucleotides (Figure 5c and Figure S7). MII = 0% corresponded to the area of lung tissue metastases in the group treated with μ-Scr-ON, while MII = 100% mirrored the complete absence of metastases (Figure 5c and Figure S7). Analysis showed that µ-oligonucleotides applied both as monotherapy and in combinations exhibited profound inhibition of internal lung metastases formation: on average, the mean MII value was 65% for pairs μ-21-ON/μ-155-ON and μ-17-ON/μ-155-ON; 70% and 75% for μ-155-ON alone and triple cocktail μ-21-ON/μ-17-ON/μ-155-ON, respectively; and slightly higher (81–85%) for μ-21-ON, μ-17-ON, and μ-21-ON/μ-17-ON (Figure 5c and Figure S7).

Despite considerable distinctions in the number of metastases and MIIs in the groups treated with oligonucleotides, there were no statistically significant differences between the effect of oligonucleotides used either separately or in cocktails (Figure 5 and Figure S7). However, the data obtained show that μ-21-ON, both as monotherapy and in combination with μ-17-ON or μ-155-ON, exhibited the highest suppression of melanoma metastatic activity, which correlates with in vitro data.

### 3.6. The Effect of Anti-miRNA Mono- and Combination Therapy with µ-Oligonucleotides on Lymphosarcoma RLS_40_ Tumor Growth (Ex Vivo Experiment)

The antitumor effect of µ-oligonucleotides was evaluated by measuring the size of the RLS_40_ primary tumors, developed in mice from RLS_40_ cells transfected ex vivo with µ-oligonucleotides.

**Figure 6:**
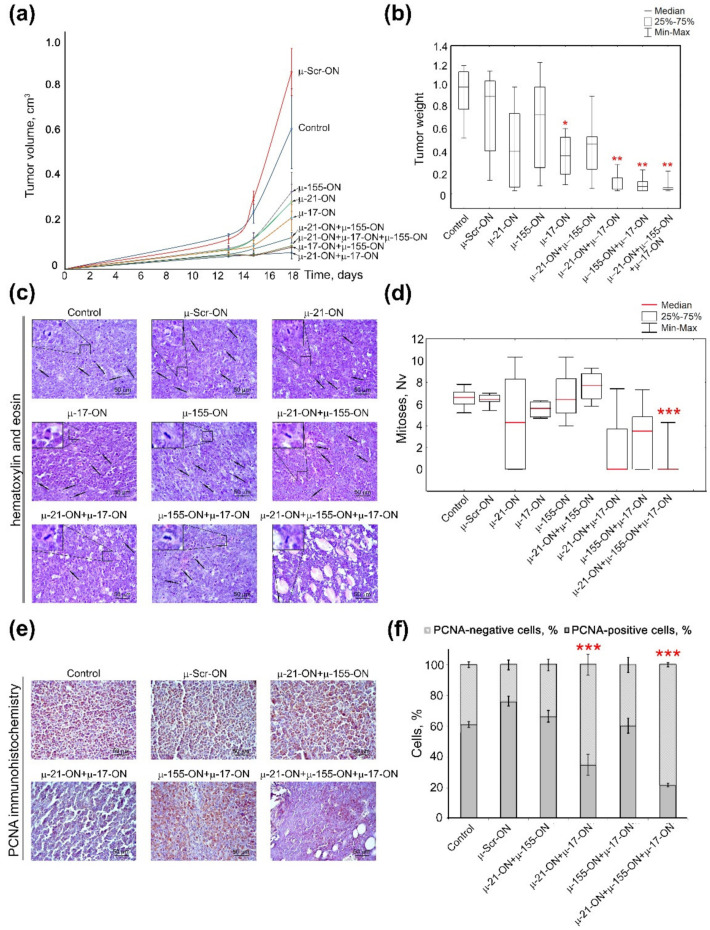
Antitumor effect of miRNA-targeted µ-oligonucleotides in the lymphosarcoma RLS_40_ model. RLS_40_ cells were transfected ex vivo with µ-ONs separately, in pairs or with cocktails of three µ-ONs (total µ-ONs concentration was 150 nM) in complex with Lipofectamine™2000 followed by implantation in mice. (**a**) Dynamics of RLS_40_ tumor growth (n = 10 mice per group). Control—mice injected with intact RLS_40_. (**b**) Average tumor weight in groups. (**c**) Typical images of tumor sections after hematoxylin and eosin staining. Magnification ×400. Bar corresponds to 50 μm. Mitoses are indicated by arrows. Typical examples of individual mitotic events are shown with magnification ×1000 in the upper left corner. (**d**) Morphometric analysis of mitotic activity of tumors in group. Nv—numerical density. (**e**) Typical images of tumor sections in groups after immunohistochemical staining with PCNA monoclonal antibodies. Magnification ×400. (**f**) Morphometric analysis of PCNA-positive and -negative cells in tumor tissue in groups. Red-colored symbols mark statistically reliable differences: *—with *p*-value ˂ 0.05 and **—with *p*-value < 0.0001 from μ-Scr-ON; ***—with *p*-value range from <0.0001 to ˂0.05 from other groups. Data were statistically analyzed using one-way ANOVA with post hoc Tukey test.

In contrast to the melanoma B16 metastatic model, the lymphosarcoma RLS_40_ model with the primary tumor node showed the clear benefit of combinative antitumor therapy in comparison with monotherapy with µ-oligonucleotides (Figure 6). Analysis of tumor growth dynamics showed that μ-155-ON, μ-21-ON and μ-17-ON exhibited significant 2.6-, 2.9- and 3.8-fold inhibition of tumor growth in comparison with µ-Scr-ON (Figure 6a); however, evaluation of average tumor weights for these groups showed that μ-155-ON did not exert any reliable antitumor effect and μ-21-ON and μ-17-ON provided 1.6- and 2-fold inhibition of tumor weight, respectively. Unfortunately, due to the large scatter of data in these groups, only μ-17-ON demonstrated a statistically reliable decrease in tumor weight (Figure 6b).

All studied combinations of µ-oligonucleotides showed more potent delay of tumor growth both in terms of tumor volume (dynamics) and tumor weight. According to the dynamics of tumor growth, μ-21-ON/μ-155-ON exhibited a more than 6-fold decrease in tumor volume (Figure 6a). The pair μ-17-ON/μ-155-ON and triple mix μ-17-ON/μ-155-ON/μ-21-ON provided a similar 9-fold decrease in tumor volume, whereas the most extensive effect was reached by the pair μ-21-ON/μ-17-ON, exhibiting a 13-fold reduction in tumor volume (Figure 6a). Considering average tumor weight, it was found that combinative application of μ-21-ON/μ-155-ON did not provide any statistically significant inhibition of tumor growth (Figure 6b), whereas pairs μ-21-ON/μ-17-ON and μ-17-ON/μ-155-ON promoted 7.4- and 8.4-fold diminishment of tumor weight, respectively (Figure 6b). The most potent antitumor effect was observed for the cocktail μ-17-ON/μ-155-ON/μ-21-ON that caused a 9-fold decrease in tumor weight, in comparison with the control µ-Scr-ON (Figure 6b).

The data obtained suggest that inhibition of miR-17 by single μ-17-ON oligonucleotide or in combination with μ-21-ON and μ-155-ON represent an efficient and perspective strategy for suppression of lymphosarcoma RLS_40_ solid tumor growth.

Histological analysis of tumor nodes showed that RLS_40_ tumors were represented by large monomorphic atypical lymphoid cells with a high mitotic rate and proliferative activity (Figure 6c,d). Microscopic examination of the tumor tissue revealed foci of necrosis, hemorrhages, and inflammatory infiltration on the border of necrosis. It should be noted that such destructive alterations were observed only in the groups with relatively big primary tumors, including the Control, µ-Scr-ON, and groups subjected to monotherapy with μ-17-ON, μ-155-ON or μ-21-ON, whereas tumor nodes from mice, treated with µ-ONs cocktails showed no signs of destructive changes.

Although monotherapy with each miRNA-targeted oligonucleotide—μ-17-ON, μ-155-ON or μ-21-ON—promoted a more than 2.5-fold delay of tumor growth, morphometric analysis of tumor sections showed that ex vivo transfection of RLS_40_ cells with these oligonucleotides used alone did not affect the mitotic potential of RLS_40_ lymphosarcoma cells transplanted into the mice (Figure 6c,d). Transfection with cocktails μ-21-ON/μ-155-ON and μ-155-ON/μ-17-ON showed no decrease in the mitotic index of RLS_40_ cells as well (Figure 6c,d). However, the combination μ-21-ON/μ-17-ON provided a more than 3-fold decrease in the numerical density of mitoses in tumor tissue in comparison with µ-Scr-ON (Figure 6c,d). The most potent antimitotic effect was exhibited by the triple cocktail μ-17-ON/μ-155-ON/μ-21-ON, providing 12- and 10.8-fold diminishment of mitosis numerical density in comparison with control and µ-Scr-ON-treated tumors, respectively (Figure 6c,d).

In order to assess the proliferative activity of implanted tumor cells, the tumor tissue sections were analyzed for the presence of Proliferating cell nuclear antigen (PCNA), which represents one of the key participants in DNA replication and subsequent cell division [46]. Immunohistochemical staining of RLS_40_ tumor sections with PCNA-specific primary antibodies demonstrated that 61.1 ± 1.9% and 76.3 ± 3.2% of tumor cells were PCNA-positive in the control and µ-Scr-ON groups, respectively (Figure 6e,f). Ex vivo treatment of tumor cells with pairs μ-21-ON/μ-155-ON and μ-155-ON/μ-17-ON did not affect the proliferative potential of RLS_40_ tumors (Figure 6e,f), while for cocktails μ-21-ON/μ-17-ON and μ-17-ON/μ-155-ON/μ-21-ON, the number of PCNA-positive cells reached 35.0 ± 6.9% and 21.8 ± 1.3%, respectively, meaning that more than a 2–3-fold decrease in the proliferative activity of tumor cells in comparison with controls was achieved (Figure 6e,f). It should be stressed that obtained data were in complete concordance with the aforementioned results of antimitotic studies for combined cell treatment with µ-oligonucleotides.

### 3.7. Efficiency of Anti-miRNA Therapy with µ-Oligonucleotides on Lymphosarcoma RLS_40_ Tumor Growth In Vivo

As it was the most potent according to ex vivo experiments, the triple combination µ-21-ON/µ-17-ON/µ-155-ON was chosen for in vivo study of the antitumor potential of combinative therapy. In these experiments, lymphosarcoma RLS_40_ cells were intramuscularly implanted into the mice thigh to initiate tumor formation; starting from day 4 of tumor development, oligonucleotides were peritumorally injected to mice (Figure 4b). Since RLS_40_ cells are characterized by high abundance of folic acid receptors on the surface [47], folate-equipped liposomes F were used for targeted delivery of µ-oligonucleotides into the tumor [31]. According to preliminary flow cytometry data, these liposomes provided efficient delivery of µ-oligonucleotides into RLS_40_ cells (Figure S8). The potency of combinative treatment was estimated relatively to the control µ-Scr-ON and monotherapy with µ-21-ON, which has already established itself as a potent antitumor agent in vivo [25]. The compounds were peritumorally administered to mice at the final concentration 10 µg per mice, since µ-oligonucleotides in this concentration were recently shown to exhibit high antitumor effect in a xenograft model of human epidermoid carcinoma KB-8-5 [25]. The concentration of each oligonucleotide in the cocktail was equal to 3.3 µg per mice. In total, three injections were made with a 3-day interval (Figure 4b).

**Figure 7:**
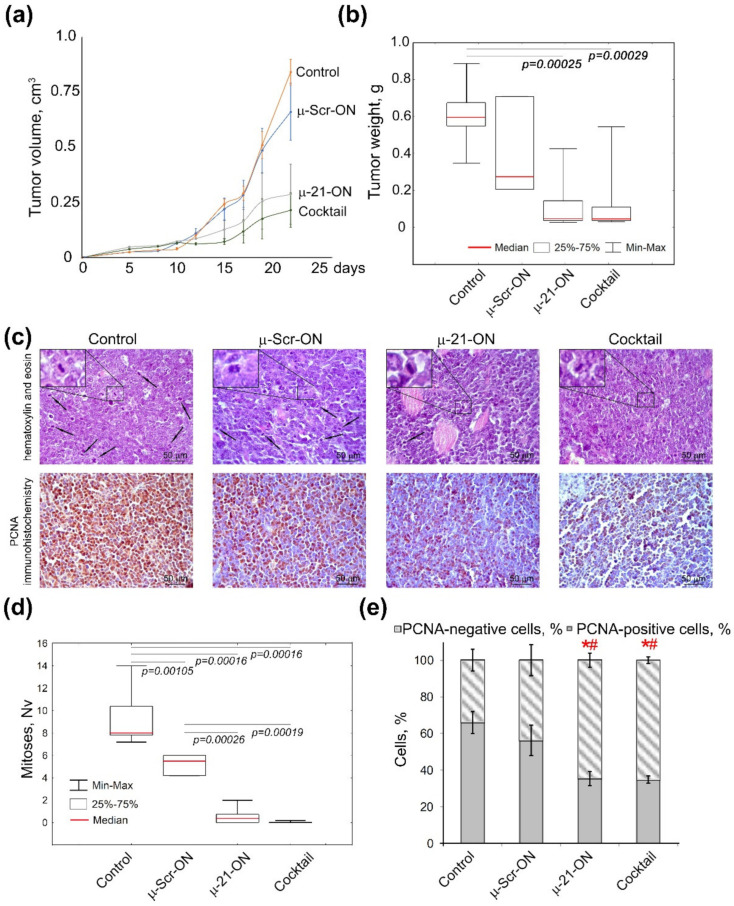
Effects of miRNA-targeted treatment with μ-oligonucleotides on lymphosarcoma RLS_40_ tumor in vivo. (**a**) Dynamics of RLS_40_ tumor growth (n = 10 mice per group). Folate-equipped liposomes F were used as a delivery agent. Control—group treated with liposomes F in the absence of oligonucleotide. µ-Scr-ON, µ-21-ON—groups treated with µ-Scr-ON and µ-21-ON, respectively, pre-complexed with liposomes F at N/P ratio 4/1 at a dose 10 μg per mice. Cocktail—group of mice treated with cocktail of µ-21-ON, µ-17-ON and µ-155-ON at a dose 3.3 μg per mice for each µ-ON. (**b**) Average tumor weight in groups at day 24. (**c**) Typical images of tumor sections after hematoxylin and eosin staining followed by immunohistochemical staining with PCNA monoclonal antibodies. Magnification ×400. Bar corresponds to 50 μm. Mitoses are indicated by arrows. Typical examples of individual mitotic events are shown with magnification ×1000 in the upper left corner. (**d**) Morphometric analysis of mitotic activity of tumor tissue in groups. Nv—numerical density. (**e**) Morphometric analysis of tumor tissue with PCNA-positive and negative cells counting. Red symbols (*#) mark statistically reliable differences from control group and a group treated with μ-Scr-ON with *p*-value ˂ 0.05. Data were statistically analyzed using one-way ANOVA with a post hoc Tukey test.

Analysis of the dynamics of RLS_40_ growth showed that miRNA-targeted µ-oligonucleotides exhibited considerable delay of tumor growth in mice: triple peritumoral injections of µ-21-ON led to an almost 3-fold decrease in tumor volume as compared to µ-Scr-ON (Figure 7a). The cocktail µ-21-ON/µ-17-ON/µ-155-ON provided an even more potent antitumor effect, leading to a 4-fold decrease in average tumor volume (Figure 7a). Comparison of tumor weight showed that both µ-21-ON and triple combination provided a 5-fold decrease in tumor weight relative to the control (Figure 7b). However, due to the significant scatter of data in these groups, a statistically reliable difference in the antitumor effects between the groups that received mono- or combinative treatment with µ-oligonucleotides was not observed.

Morphometric evaluation of RLS_40_ tumor sections revealed that peritumoral injections of µ-21-ON led to remarkable decreases in the numerical density of mitoses in tumor tissue by the factor of 18.4 and 10.4 compared with the control and µ-Scr-ON-treated groups, respectively. Moreover, treatment with the cocktail µ-21-ON/µ-17-ON/µ-155-ON exhibited extremely potent antimitotic effects, promoting up to a 230 times decrease in the mitotic index corresponding to almost complete inhibition of mitotic activity in tumor node (Figure 7c,d).

Morphometric analysis of immunohistochemical images showed that RLS_40_ tumor tissue without treatment (control) and tumors injected with control oligonucleotide µ-Scr-ON has 66 ± 6.1% and 56.2 ± 8.3% of PCNA-positive cells, respectively (Figure 7c,e). Treatment of mice with both µ-21-ON and µ-21-ON/µ-17-ON/µ-155-ON led to an almost 2-fold decrease in the number of PCNA-positive tumor cells compared with the control groups, being equal to 35% of PCNA-positive cells (Figure 7c,e).

### 3.8. Toxicity of Anti-miRNA Combinative Treatment in Mice

For assessment of anti-miRNA treatment toxicity in mice, histological analyses of livers and kidneys of healthy, control RLS_40_ lymphosarcoma-bearing mice, and those who received anti-miRNA-ONs were carried out. Table 1 and Figure S5 show that the development of RLS_40_ lymphosarcoma in the control and µ-Scr-ON-treated groups causes significant toxic effects onto the liver represented by an almost 4-fold increase in the total destructive changes in the liver parenchyma due to dystrophies and necrosis equally compared to healthy animals (Table 1, Figure S9). However, therapy with µ-21-ON led to an almost 1.5-fold decrease in the necrosis-occupied fraction of the liver compared to controls; it had no reliable influence on total destructive changes in the liver (Table 1, Figure S9). In turn, in the group that received the cocktail µ-21-ON/µ-17-ON/µ-155-ON, a 2-fold decrease in the total destructive changes in the liver parenchyma was observed, manifested in the reduction in both dystrophies and necrosis compared to controls (Table 1, Figure S9). Assessment of the regenerative potential of the liver revealed 2.1- and 1.4-fold increases in the number of binuclear hepatocytes in the livers of tumor-bearing mice treated with µ-21-ON and cocktail compared to the control animals (Table 1, Figure S9). Morphometric analysis of kidneys revealed no toxic effects of both RLS_40_ lymphosarcoma growth and anti-miRNA-ON therapy.

### 3.9. Bioinformatic Analysis of Possible Molecular Interactions Underlying the Effects of Mono- and Combination Therapy with µ-Oligonucleotides

**Figure 8:**
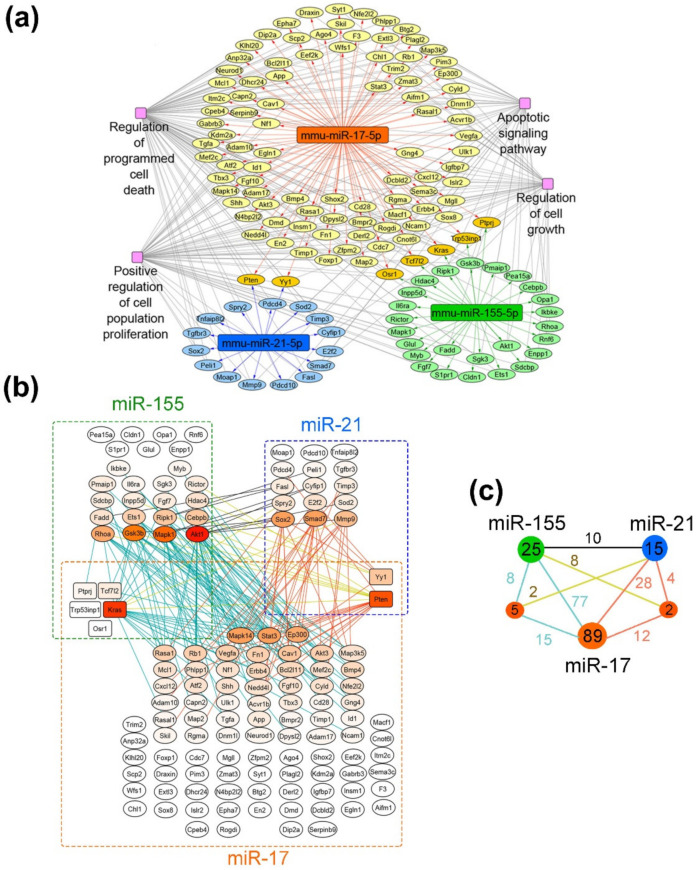
Proliferation-related targets and possible molecular interactions underlying the effect of miRNA-targeted µ-oligonucleotides on proliferation. (**a**) Regulome of miRNAs and their target genes reconstructed using the miRTarBase 8.0 (*Mus musculus*) database. The analysis was carried out using the CyTargetLinker plugin in Cytoscape. (**b**) Gene–gene interactions between miR-17, miR-21 and miR-155 targets analyzed using the String database. (**c**) The general scheme, depicting the number of gene–gene interactions between individual and common target genes of miR-17, miR-21 and miR-155. The numbers in green, dark blue and orange circles are related to the number of individual target genes regulated by miR-155, miR-21 and miR-17, respectively. The numbers in smaller terracotta circles are related to the number of common target genes regulated by both miRNAs in miR-155/miR-21 and miR-21/miR-17 pairs. Numbers above the junctions relate to the interactions between both individual miRNA targets as well as individual and common targets managed by miRNAs: black—between individual targets of miR-155 and miR-21; terracotta—between individual targets of miR-17 and miR-21 as well as their interconnections with common targets of these miRNAs; blue—between individual targets of miR-17 and miR-155 as well as their interconnections with common targets of these miRNAs; yellow—between common targets regulated by miR-21/miR-17 and individual targets of miR-155 as well as common targets regulated by miR-155/miR-17 and individual targets of miR-21, respectively.

To reveal the potential molecular mechanisms underlying the antitumor effects of evaluated miRNA-targeted therapy with µ-oligonucleotides, bioinformatics analysis of miR-17-, miR-21-, and miR-155-associated regulomes was performed. The target genes of each miRNA in the murine transcriptome were reconstructed using the miRTarBase database, which contains information solely about experimentally validated miRNA-regulated targets. The whole regulatory network “miRNA-target genes” is presented in the Supplementary Materials (Figure S10). The obtained regulome was used for functional analysis in order to deprive the functional terms associated with cell proliferation (Figure 8) and metastasis (Figure S11). Gene–gene interconnection analysis was carried out using the String database, in order to evaluate the molecular interactions of targets managed by different miRNAs.

As can be seen from the gene network in Figure 8a, miR-17 regulates the leading number of genes, involved in cell proliferation: 96 out of 414 validated targets (24%). At the same time, miR-155 and miR-21 possess a significantly lower number of targets, associated with cell growth—30 out of 87 for miR-155 (35%) and 17 out 37 for miR-21 (46%). It should be noted that miRNAs have several targets in common, including five genes for miR-17/miR-155 (*Osr1*, *Tcf7I2*, *Kras*, *Trp53inp1* and *Ptprj*) and two genes for miR-17/miR-21 (*Pten* and *Yy1*) (Figure 8a,b).

Considering the effects of monotherapy with µ-oligonucleotides targeted to miR-17, miR-21 and miR-155, it can be assumed that the efficiency of treatment is associated with the expression level of miRNA itself and the number of individual function-related genes managed by this miRNA. In melanoma B16 cells, the in vitro effect of µ-21-ON was higher than µ-17-ON and µ-155-ON, which might be explained by the greater number of target genes closely associated with proliferation (about 46% of the total number). In contrast, in the RLS_40_ model ex vivo, the most potent tumor growth delay was shown for µ-17-ON, whereas µ-21-ON and µ-155-ON exerted no statistically reliable antitumor effect. This observation can be explained by (1) higher expression of miR-17 and other members of the miR-17~92 family in lymphoid cells and, in particular, lymphosarcoma, which was repeatedly confirmed in previous studies [48,49] and (2) the large number of individual proliferation-associated targets of miR-17, in contrast to miR-21 and miR-155 (Figure 8b).

The observed synergetic action of miRNA-targeted combinations might be realized by (i) the enhanced modulation of common molecular targets of selected miRNAs; and (ii) by concurrent influence on all function-related mRNA managed individually by two or three miRNAs, leading to reinforcing of the effect due to feedback loops and crosstalks between downstream targets. Considering the gene–gene interconnection study, it becomes obvious that for the miR-17/miR-155 pair, synergetic effects can be realized by modulation of common protein targets *Tcf7I2* and *Kras*, which interact with various targets managed individually by miR-17 and miR-155 (in total, 23 interactions) (Figure 8b,c). Moreover, the multiple interconnections between the targets such as *Akt1*, *Mapk1*, *Gsk3b*, *Rhoa*, *Cebpb*, *Ripk1*, *Ets1*, *Fadd*, etc., directly managed by miR-155, and *Mapk14*, *Stat3*, *Rasa1*, *Rb1*, *Vegfa*, *Fn1*, *Cav1*, *Ep300*, etc., being the first-order targets of miR-17, also supplied the effect of this combination (in total, 77 interactions) (Figure 8b,c). For miR-17/miR-21, the common nodal target is *Pten*, which is an established potent tumor suppressor, mostly known as a regulator of the PI3K/Akt pathway and as possessing multiple interconnections with miR-17- and miR-21-regulated targets, accounted for 16 interconnections in total (Figure 8b,c). *Pten*-mediated antiproliferative effect is additionally enhanced by interconnections between individual targets of miR-17 listed previously and direct targets of miR-21 such as *Sox2*, *Smad7*, *E2f2*, *Sod2*, *Timp3*, *Fasl*, *Mmp9*, etc. (28 interconnections in total) (Figure 8b,c). In turn, miR-21/miR-155 has no common protein targets to manage; however, more than a dozen of the gene–gene pair intersections in managed genetic networks were found: between *Akt1*, *Mapk1*, *Gsk3b*, *Rhoa*, *Fadd*, *Ets1*, *Ripk1*, *Cebpb*, etc. managed by miR-155 and *Sox2*, *Smad7*, *Mmp9*, *Spry2*, *E2f2*, *Sod 2*, etc., regulated by miR-21 (10 interconnections, in total) (Figure 8b,c). The data obtained for this pair suggest that synergetic effects are provided by simultaneous modulation of all proliferation-related target genes of these miRNAs in sum, mutually reinforced by downstream intergenic interactions (Figure 8b). The effect of the triple cocktail miR-17/miR-21/miR-155 combines all the interactions observed for oligonucleotide pairs and is, moreover, strengthened by the interplaying of common genes, capable of mediating the interactions between all three selected miRNAs, resulting in a total of 164 interconnections (Figure 8b,c).

In the case of regulome, controlling the metastatic potential of tumor cells (Figure S11), the miR-17-targeted gene pool also turned out to be the most enriched with 67 targets out of 414 genes (16%) in comparison with miR-155 (16 out of 67) and miR-21 (14 out of 37). Several common targets of miR-17 with miR-155 and miR-21 were defined, including *Trp53inp1*, *Ptprj* and *Pten*, respectively (Figure S11b). For the miR-17/miR-155 pair, the common protein target is *Ptprj*. Since *Ptprj* interacts only with a few individual targets such as *Gsk3b* regulated by miR-155 and *Vegfa* and *Ptprj* managed by miR-17, it seems that this combination provides its effect mostly through interconnections between individual targets of miRNAs, including *Akt1*, *Mapk1*, *Gsk3b*, and *Rhoa*, driven by miR-155, and *Mapk14*, *Stat3*, *Cdh2, Cav1*, *Ptpn11*, etc., that represent direct targets of miR-17 (about 52 interconnections in total) (Figure S11b,c). For miR-17/miR-21, the common nodal target is again *Pten*, which does not have any interactions with miR-21 molecular targets, but possesses the wide net of interconnections with miR-17-directed targets, including *Mapk14*, *Stat3*, *Cdh2*, *Cav1*, *Ptpn11*, *Fn1*, *Akt3*, *Erbb4*, etc. (about 35 interconnections in total) (Figure S11b,c). Similar to the proliferation bioinformatic study, the effect of the triple cocktail miR-17/miR-21/miR-155 combines all the interactions observed for oligonucleotide pairs and is additionally improved by multiple interactions of *Pten* with individual targets of miR-155, resulting in 107 interactions in total (Figure S11b,c). miR-21/miR-155 again has no common protein targets to regulate and may promote their antimigration effect through various gene–gene pair intersections between miR-155-related targets, such as *Akt1*, *Mapk1*, *Gsk3b*, *Rhoa*, and *Fadd*, and miR-21-directed genes, including *Mmp9*, *Sox2*, *Spry2*, *Reck*, *Map3k1*, *Map3k2*, *Tns1* and *Sod 2* regulated by miR-21 (about 13 interconnections in total) (Figure S11b,c).

**Figure 9:**
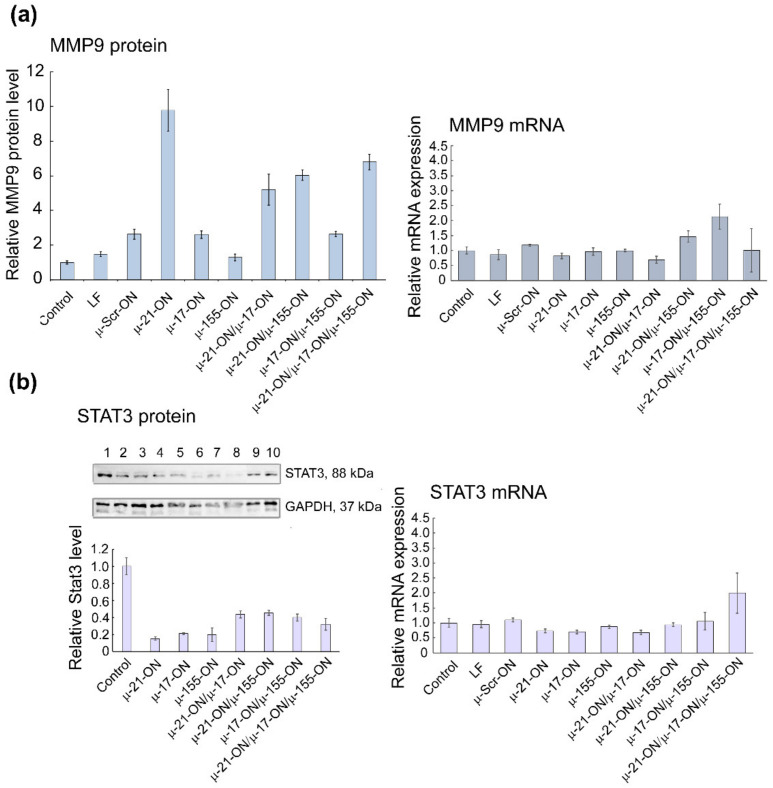
Analysis of mRNA and protein levels of genes, regulated by miR-21, miR-17 and miR-155 in murine melanoma B16 cells after treatment with µ-oligonucleotides. (**a**) Relative level of MMP9 protein measured by flow cytometry analysis and relative expression of MMP9 mRNA measured by qPCR 72 h after mono- and combinative treatment with µ-oligonucleotides. (**b**) Relative level of STAT3 protein measured by Western blot analysis and relative expression of *STAT3* mRNA measured by qPCR 72 h after mono- and combinative treatment with µ-oligonucleotides. The numbers above the Western blot image correspond to the following experimental groups: 1—Control (intact B16 cells); 2—B16 cells incubated with Lipofectamine2000^TM^; 3, 4, 5, and 6—cells incubated with singular oligonucleotides µ-Scr-ON, µ-21-ON, µ-17-ON, and µ-155-ON, respectively, in the 150 nM concentration; 7—cells incubated with µ-21-ON/µ-17-ON pair (75 nM of each ON); 8—cells incubated with triple combination µ-21-ON/µ-17-ON/µ-155-ON (50 nM of each ON); 9 and 10—cells treated with pairs µ-21-ON/µ-155-ON and µ-155-ON/µ-17-ON, respectively (75 nM of each ON). The level of STAT3 protein was normalized to the level of GAPDH protein.

**Figure 10:**
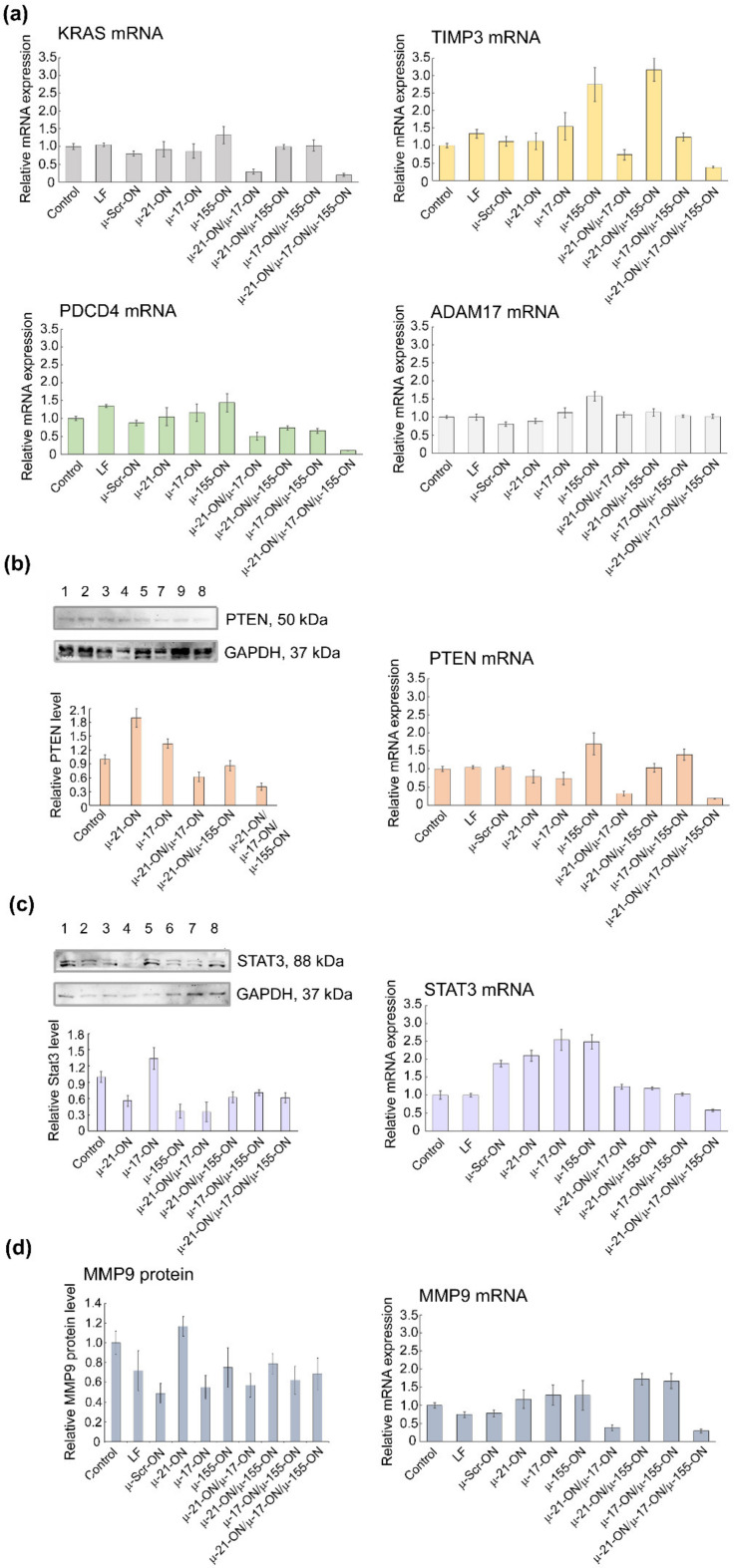
Analysis of mRNA and protein levels of genes, regulated by miR-21, miR-17 and miR-155 in murine lymphosarcoma RLS_40_ cells after treatment with µ-oligonucleotides. (**a**) Relative expression of *KRAS*, *TIMP3*, *PDCD4*, and *ADAM17* mRNAs 72 h after transfection of cells with µ-oligonucleotides alone and in combination, measured by qPCR. (**b**) Relative level of PTEN protein measured by Western blot analysis and relative expression of *PTEN* mRNA measured by qPCR 72 h after mono- and combinative treatment with µ-oligonucleotides. (**c**) Relative level of STAT3 protein measured by Western blot analysis and relative expression of *STAT3* mRNA measured by qPCR 72 h after mono- and combinative treatment with µ-oligonucleotides. The numbers above the Western blot image correspond to the following experimental groups: 1—Control (intact RLS_40_ cells); 2—RLS40 cells incubated with Lipofectamine2000^TM^; 3, 4, 5, and 6—cells incubated with singular oligonucleotides µ-Scr-ON, µ-21-ON, µ-17-ON, and µ-155-ON, respectively (150 nM); 7—cells incubates with the µ-21-ON/µ-17-ON pair (75 nM of each ON); 8—cells incubated with the triple combination µ-21-ON/µ-17-ON/µ-155-ON (50 nM of each ON); 9—cells treated with pair µ-21-ON/µ-155-ON (75 nM of each ON). (**d**) Relative level of MMP9 protein measured by flow cytometry analysis and relative expression of MMP9 mRNA measured by qPCR 72 h after mono- and combinative treatment with µ-oligonucleotides. The level of STAT3 and PTEN protein was normalized to the level of GAPDH protein. Expression of mRNAs was normalized to the expression of housekeeping genes *HPRT1* and *GAPDH*.

The initial validation of bioinformatic data was carried out in both tumor cell lines and included the evaluation of *KRAS*, *PTEN*, *TIMP3*, *PDCD4*, *MMP9*, *STAT*3 and *ADAM17* mRNA levels by qPCR, quantification of Pten and Stat3 protein levels by Western blot analysis, and measurement of Mmp9 protein level by flow cytometry (Figure 9, Figure 10 and Figure S12). A brief description of investigated gene targets is given in Table S2 [50,51,52,53,54,55,56,57,58,59,60,61,62].

For B16 cells, investigation of mRNA levels by qPCR demonstrated no statistically reliable changes in target gene expression 72 h after transfection (Figure S12), which can be explained by the steric block of mRNAs by corresponding miRNAs rather than degradation. At the protein level, significantly, up to 6- to 10-fold increases in Mmp9 protein, regulated by miR-21, were detected after transfection with either singular µ-21-ON or combinations, containing this oligonucleotide (Figure 9a). In addition, an about 5-fold decrease in oncogenic Stat3 protein, directly managed by miR-17, was observed for all singular oligonucleotides—µ-21-ON, µ-17-ON and µ-155-ON—and about 2-fold for their paired or triple combinations (Figure 9b).

In RLS_40_ cells, according to qPCR data, a decrease in mRNA levels of the nodal gene *KRAS* (regulated by miR-155/miR-17) and *PTEN* (regulated by miR-21/miR-17) after treatment with µ-21-ON/µ-17-ON and µ-21-ON/µ-17-ON/µ-155-ON was detected (Figure 10a). In addition, differences in the mRNAs of individual gene targets were demonstrated, including a decrease in *TIMP3*, and *PDCD4* (regulated by miR-21) mRNA level after transfection with µ-21-ON/µ-17-ON and µ-21-ON/µ-17-ON/µ-155-ON and an increase in *TIMP3* expression in the µ-155-ON- and µ-21-ON/µ-155-ON-treated groups (Figure 10a).

Western blot analysis showed that Pten protein level (managed by both, miR-21 and miR-17) increased almost 2-fold after treatment with singular µ-21-ON, but significantly diminished after treatment with all combinations containing µ-21-ON, which correlates well with qPCR data and may indicate the presence of complex downstream interactions and feedback loops between targets (Figure 10b). Both, mRNA and protein levels of the STAT3 gene were increased after treatment with µ-17-ON, and were diminished in the case of combined applications (Figure 10c). For the *MMP9* gene, a significant decrease in the mRNA level was observed in groups treated with µ-21-ON/µ-17-ON and µ-21-ON/µ-17-ON/µ-155-ON, but no statistically reliable changes in protein levels were detected (Figure 10d).

The obtained data obviously show that the molecular effects of combinations of miRNA-targeted oligonucleotides are realized due to the impact of a number of target genes predicted by bioinformatics analysis. It can be assumed that in the murine melanoma B16 model, the effect of µ-oligonucleotides is more likely provided through the influence on the individual gene targets of miRNAs (MMP9/miR-21, STAT3/miR-17); however, in the lymphosarcoma RLS_40_ model, treatment with combinations may cause mRNA and protein functioning alterations for both nodal genes (KRAS/miR-17/miR-155; PTEN/miR-21/miR-17) and individual targets (TIMP3, MMP9 or PDCD4/miR-21, STAT3/miR-17).

Our in vitro and ex vivo studies clearly show that combinations µ-21-ON/µ-17-ON, µ-21-ON/µ-155-ON and µ-21-ON/µ-17-ON/µ-155-ON provide a synergistic effect on the proliferation and migration of tumor cells (Figure 2, Figure 3 and Figure 6). The further validation demonstrated the association of these effects with changes at the transcriptomic and proteomic levels (Figure 9, Figure 10 and Figure S12). Although the presented results do not provide sufficient information to make an unambiguous conclusion about the nature of the effect—synergism/antagonism—of combination therapy with miRNA-targeted oligonucleotides at the proteome level of tumor cells, it is obvious that the degree and kinetics of the decrease in miRNA levels (Figures S5 and S6), the effect on specific genes, and the degree and nature of changes in their expression (positive/negative effect) differ markedly for mono- and combination therapy with oligonucleotides (Figure 9, Figure 10 and Figure S12). These results suggested the complex intracellular intergenic mechanisms of interactions due to the simultaneous suppression of two or three miRNAs, leading to a pronounced antitumor effect.

It should be noted that the proposed bioinformatics model was reconstructed based on a large number of studies, including all experimentally validated targets of selected miRNAs in mouse cells of various histological types and degrees of oncotransformation. Such gene networks can give us just a general overview of the possible molecular interactions, providing the effects of individual µ-oligonucleotides targeted to miR-17, miR-21 and miR-155 and their combinations. The actual direct gene targets, their total number for each miRNA, degree of involvement in the carcinogenesis processes, as well as the gene–gene interconnections between targets in the miRNA regulomes should be evaluated for each tumor model studied with the possible involvement of omics technologies.

## 4. Discussion

Interest in oligonucleotide agents aimed at the modulation of activity of regulatory non-coding RNAs, especially miRNAs, is rapidly growing nowadays. Successful application of single miRNA-based therapeutics in various tumor models allowed us to suggest that combination of such drugs might be a beneficial strategy for neoplasia treatment in comparison with single-agent management.

The outcome of the therapy to a great extent depends on the choice of miRNA target, which includes large-scale analysis of key functional parameters, such as miRNA expression level in specific histological types of tumor cells as well as the involvement of miRNA and its downstream targets in tumor-associated processes. Depending on the tasks, researchers can select the miRNA target, which is either a function-specific actor, managing mostly one cellular process or a potent, multifunctional regulator, which controls numerous downstream molecules. During the last seven years, about six dozen of combinations based on anti-miRNA ONs and miRNA mimics have been designed and investigated, targeted to more than forty different miRNAs [13,16,63,64,65,66].

However, in spite of the amazing progress in the development of miRNA-targeted oligonucleotide-based combinations, the vast majority of designed cocktails are characterized by the additive nature of interactions. Such additive action was, for instance, observed for the anti-miR-17 and anti-miR-20a combination that exhibited a 70% decrease in lung cancer cell proliferation, while the effect of individual compounds was about 60% [67]. Only limited combinations, usually consisting of anti-miR-ON or miRNA-mimic and chemotherapeutic agent, exhibited synergetic effects ex vivo or in vivo. For instance, a 6-fold decrease in tumor growth rate was observed for the miR-205 mimic and gemcitabine in a pancreatic cancer model ex vivo and for let-7b mimic and paclitaxel in a non-small cell lung cancer model in vivo [13,19]. However, despite the high potency of this type of combination (miRNA-targeted oligonucleotide + chemotherapy), their action is usually accompanied by significant toxicity and diminished specificity, which is why cocktails containing several miRNA-targeted oligonucleotides might be beneficial as a specific and low-toxicity therapeutic remedy for the treatment of oncological diseases.

Herein for the first time, we carried out an investigation on the efficiency of combinative therapy applying antisense oligonucleotides targeted to miR-21, miR-17 and miR-155 in two types of two tumor models with completely different histogenesis and characteristics, including highly aggressive melanoma B16 originating from neuroectoderm and lymphosarcoma RLS_40_ that represents hematological multidrug-resistant oncological disease. Summarizing the data obtained, among all three miRNA-targeted oligonucleotides, the weakest antiproliferative effect on both models was observed for µ-155-ON (Figure 2, Figure 3, Figure 5 and Figure 6), whereas the potency of µ-21-ON and µ-17-ON was higher but different in these cell lines: in melanoma B16, a more pronounced antiproliferative effect was demonstrated by both µ-21-ON and µ-17-ON (Figure 2, Figure 3 and Figure 5), while, in the RLS_40_ model, µ-17-ON was more effective (Figure 6).

Further complex analysis of the data unequivocally showed that the therapeutic potential of µ-21-ON, µ-17-ON and µ-155-ON was significantly enhanced in vitro and in vivo when applied in combination (Figure 2, Figure 3 and Figure 6). Cocktails µ-21-ON/µ-17-ON and µ-21-ON/µ-155-ON were able to synergistically suppress two key cellular processes at a time—proliferation and migration; synergism was already achieved at an oligonucleotide concentration of 25 nM and was observed in the entire dose range studied (Figure 2 and Figure 3). It is one of the rarest examples of combinations exhibiting simultaneous synergetic inhibition of several carcinogenesis processes. Earlier, this bifunctional synergy was only demonstrated for the cocktail of oligonucleotides targeted to miR-183, miR-182 and miR-96, which provided 5-fold proliferation inhibition and complete suppression of colony formation of colon cancer cells [21].

Ex vivo experiments with multidrug-resistant lymphosarcoma RLS_40_ also demonstrated a clear advantage of combinative therapy over monotherapy: the antitumor potential of µ-21-ON and µ-17-ON increased 3–4 times when applied as a pair or a triple combination, leading to almost 13-fold inhibition of tumor growth (Figure 6). The most highly effective in both tumor models were combinations simultaneously containing µ-21-ON and µ-17-ON, including the µ-21-ON/µ-17-ON pair and the triple cocktail µ-21-ON/µ-17-ON/µ-155-ON (Figure 5, Figure 6 and Figure 7 and Table S3). Moreover, the terrific benefit of a triple cocktail µ-21-ON/µ-17-ON/µ-155-ON administered in vivo was observed, showing a record decrease in the tumor mitotic index, leading to almost complete suppression of cell division and growth of the primary tumor node (Figure 7 and Table S3). Moreover, this combination exhibited the significant therapeutic effect on liver parenchyma, leading to a considerable decrease in total destructive changes (Table 1). Efficiency and low toxicity, together with the protective effect on the liver of the proposed therapy, can contribute to a significant increase in the lifespan of tumor-bearing mice; however, according to bioethical guidelines for the welfare and use of animals in cancer research, such studies cannot be carried out.

It is essential to recall that the tumor type (metastatic or solid) exerts a direct influence on treatment efficiency. Indeed, the formation of metastases is the process associated with diminished adhesion, sustained survival in the bloodstream, and increased motility and colonization ability of tumor cells, whereas the growth of the primary tumor node is mostly maintained by local uncontrolled excessive tumor cell proliferation. Since then, the observed difference in the effect of µ-oligonucleotides in metastatic (B16) and solid (RLS_40_) models lies in the variations in processes affected by treatment with a major impact of oligonucleotides on motility, extravasation and adhesion in the metastatic model vs. pointwise influence on cellular proliferation in the primary tumor node. It should be mentioned that, in ex vivo studies, in order to compare the performance of mono-oligonucleotides and combinations, we made the concentration of µ-oligonucleotides uniform and chose the maximum possible total dose according to in vitro data, which exhibited a highly effective synergetic decrease in cellular proliferation and migration. Probably, the overall decrease in the total concentration of oligonucleotides upon transfection or individual concentration-related study for each combination will allow differences between mono and combinative treatment effects to be evaluated in the melanoma metastatic model. It also should be noted that enhancement of therapeutic efficiency can be achieved by adjustment of oligonucleotides doses and treatment regimen. Triple injection of µ-21-ON/µ-17-ON and µ-21-ON/µ-17-ON/µ-155-ON almost completely impeded the primary tumor growth up to day 18 after tumor cell implantation (Figure 6a and Figure 7a). It might be suggested that optimization of the injection scheme, including the increase in the number of injections and/or changes in the time interval between the injections, should significantly prolong the effect of therapy, even further reducing tumor growth rate or completely abort it.

Conducting bioinformatic analysis offered the possible molecular interactions underlying the effects of the most promising combinations. As we can see, the µ-21-ON/µ-17-ON pair might have an impact on either individual targets of each miRNA or common managed proteins (Figure 8b and Figure S11b). In general, four dozens of distinct gene–gene interactions between common and individual genes are involved in the effect of µ-21-ON/µ-17-ON pair, engaging PI3K/Akt/mTOR-signaling; Stat3-, MAPK- and TGF-β/Smad-signaling; and Pdcd4-mediated ERK-NF-κβ signaling pathways, each of which plays a crucial role in the processes of proliferation, migration and cell survival (Figure 8 and Figure S11) [68,69,70,71]. In the case of the triple cocktail, the genetic network is complemented by 120 more miRNA-regulated gene–gene interactions (Figure 8c), resulting in a manifold decrease in the mitotic activity of cells in the tumor node and pronounced protective effects on liver parenchyma in vivo that cannot be attained in the case of monotherapy.

In this study, we clearly showed that a combinative treatment strategy might provide unquestionable benefits over singular oligonucleotide agents, providing simultaneous and potent inhibition of several carcinogenesis processes at a time. A competent choice of miRNA targets for the specific histological type of malignant neoplasia will allow an excellent therapeutic remedy to be created that can become a new word in the treatment of oncological diseases.

## 5. Conclusions

This study, for the first time, elucidated the consequences of monotherapy vs. combination treatment of two highly aggressive tumor models with mesyl phosphoramidate oligodeoxynucleotides targeted to the established pro-oncogenic microRNAs miR-17, miR-21 and miR-155. We showed that silencing of two (miR-17/miR-21 or miR-155/miR-21) or three miRNAs (miR-17/miR-21/miR-155) at a time has clear advantages over downregulation of only one particular miRNA target. Moreover, a cocktail of three anti-miR µ-oligonucleotides represents the most successful strategy for antitumor treatment, since it allowed metastases formation to be significantly inhibited and tumor growth to be delayed in mice. The essential benefit of the designed triple µ-17-ON/µ-21-ON/µ-155-ON combination lies in its high-selectivity, the absence of toxic off-target effects, and its liver-protective properties, which may turn such a cocktail into a promising therapeutic instrument in the management of miRNA-associated oncological diseases.

## Figures and Tables

**Table 1:** Morphological changes in the liver tissue of healthy mice and mice with RLS_40_ lymphosarcoma without treatment (Control) and after anti-miRNA-ON administration.

	Healthy	Control	µ-Scr-ON	µ-21-ON	Cocktail
Normal hepatocytes, Vv, %	72.2 ± 1.6	30.5 ± 3.3 †	31.1 ± 3.3 †	35.9 ± 3.3 †	59 ± 3.5 †*#
Dystrophy, Vv, %	7.2 ± 1	29.8 ± 2.9 †	28.1 ± 1.9 †	31.3 ± 2.6†	13.7 ± 1.3 †*#
Necrosis, Vv, %	7.8 ± 1.3	27.8 ± 1.5 †	29.3 ± 3.3 †	21.1 ± 1.4 †*#	14.3 ± 1.6 †*#
Total destructive changes, Vv, %	15 ± 2.1	57.6 ± 3.3 †	57.3 ± 3.8 †	52.4 ± 3.6†	28 ± 2.5 †*#
Stroma, Vv, %	12 ± 1.7	11.9 ± 1.1	11.3 ± 1.2	11.5 ± 1.3	10.1 ± 1.2
Binuclear hepatocytes, Nv	4.4 ± 1.1	3.4 ± 0.7	4.7 ± 0.9	7.1 ± 1.1 †*	4.9 ± 0.7 *

Vv—volume density; Nv—numerical density; †—statistically significant differences compared to healthy animals, *p* ≤ 0.05; *—statistically significant differences compared to control, *p* ≤ 0.05; #—statistically significant differences compared to µ-Scr-ON, *p* ≤ 0.05.

## Data Availability

Not applicable.

## Supplementary Materials

The following supporting information can be downloaded at: https://www.mdpi.com/article/10.3390/cancers14184396/s1, Figure S1: Relative expression of miR-17, miR-21 and miR-155 in lymphosarcoma RLS_40_ and melanoma B16 cells; Figure S2: Dose response curves of B16 melanoma cells proliferation depending on concentration of µ-21-ON, µ-17-ON and µ-155-ON; Figure S3: Inhibition of migrative activity of melanoma B16 cells by miRNA-targeted μ-oligonucleotides; Figure S4: Antimigrative effect of oligonucleotide cocktail µ-17-ON/µ-155-ON on B16 melanoma cells; Figure S5: The level of miR-17, miR-21 and miR-155 in melanoma B16 cells after treatment with µ-oligonucleotides alone and combinations; Figure S6: The level of miR-17, miR-21 and miR-155 in lymphosarcoma RLS_40_ cells after treatment with µ-oligonucleotides alone and combinations; Figure S7: The effect of treatment with miRNA-targeted µ-oligonucleotides on formation of melanoma B16 metastases ex vivo; Figure S8: Efficiency of target delivery of FITC-labelled oligonucleotide using folate-equipped liposomes F to lymphosarcoma RLS_40_ cells measured by flow cytometry; Figure S9: Liver and kidney toxicity of miRNA-targeted treatment with μ-oligonucleotides on lymphosarcoma RLS_40_; Figure S10: Functional annotation of miRNAs targeted in the study; Figure S11: Migration-related targets and possible molecular interactions underlying the effect of miRNA-targeted µ-oligonucleotides on cells motility; Figure S12: Analysis of mRNA and protein levels of genes, regulated by miR-21, miR-17 and miR-155 in murine melanoma B16 cells after treatment with µ-oligonucleotides; Figure S13: Analysis of mRNA and protein levels of genes, regulated by miR-21, miR-17 and miR-155 in murine lymphosarcoma RLS_40_ cells after treatment with µ-oligonucleotides; Table S1: Sequences of RT and PCR primers used in the study; Table S2: The role in regulome, intracellular localization and function of genes, selected for validation of bioinformatic data by qPCR, Western blot or Flow cytometry analysis; Table S3: Comparison of µ-21-ON and µ-21-ON/µ-17-ON/µ-155-ON performance in lymphosarcoma RLS_40_ model ex vivo and in vivo.
