# Supplementary material for: Single Shot vs. Cocktail: A Comparison of Mono- and Combinative Application of miRNA-Targeted Mesyl Oligonucleotides for Efficient Antitumor Therapy
Source: Cancers (Basel). 2022 Sep 9;14(18):4396. doi: 10.3390/cancers14184396 (PMC9496860; doi:10.3390/cancers14184396)
Supplement: Supplementary file 1 [file cancers-14-04396-s001.zip › cancers-1854158-supplementary.pdf]

Supplementary materials

Single Shot vs. Cocktail: A Comparison of Mono- and Combinative Application of miRNA-Targeted Mesyl Oligonucleotides for Efficient Antitumor Therapy

Svetlana Gaponova [1], Olga Patutina [1*], Aleksandra Sen'kova [1], Ekaterina Burakova [2,3], Innokenty Savin [1], Andrey Markov [1], Elena Shmendel [4], Mikhail Maslov [4], Dmitry Stetsenko [2,3], Valentin Vlassov [1], and Marina Zenkova [1]

[1] Institute of Chemical Biology and Fundamental Medicine SB RAS, Lavrentiev's ave. 8, 630090, Novosibirsk, Russia

[2] Department of Physics, Novosibirsk State University, 1 Pirogova str., Novosibirsk 630090, Russia

[3] Sector of Plant Chemical Biology, Institute of Cytology and Genetics, SB RAS, Lavrentiev Ave. 10, Novosibirsk 630090, Russia

[4] Department of Chemistry and Technology of Biologically Active Compounds, Medical and Organic Chemistry named after N.A. Preobrazhensky, MIREA – Russian Technological University, Vernadsky Ave. 78, Moscow 119454, Russia

* Correspondence: patutina@niboch.nsc.ru; Tel.: +7-383-3635161 or +7-9231484230 (O.P.)

Table of contents:

1. Relative expression of miR-17, miR-21 and miR-155 in lymphosarcoma RLS_{40} and melanoma B16 cells
2. Dose response curves of B16 melanoma cells proliferation depending on concentration of μ-21-ON, μ-17-ON and μ-155-ON
3. Inhibition of migrative activity of melanoma B16 cells by miRNA-targeted μ-oligonucleotides
4. Antimigrative effect of oligonucleotide cocktail μ-17-ON/μ-155-ON on B16 melanoma cells
5. The level of miR-17, miR-21 and miR-155 in melanoma B16 cells after treatment with μ-oligonucleotides alone and combinations
6. The level of miR-17, miR-21 and miR-155 in lymphosarcoma RLS_{40} cells after treatment with μ-oligonucleotides alone and combinations
7. The effect of treatment with miRNA-targeted μ-oligonucleotides on formation of melanoma B16 metastases *ex vivo*
8. Efficiency of target delivery of FITC-labelled oligonucleotide using folate-equipped liposomes F to lymphosarcoma RLS_{40} cells measured by flow cytometry
9. Liver and kidney toxicity of miRNA-targeted treatment with μ-oligonucleotides on lymphosarcoma RLS_{40}
10. Functional annotation of miRNAs targeted in the study
11. Migration-related targets and possible molecular interactions underlying the effect of miRNA-targeted μ-oligonucleotides on cells motility
12. Analysis of mRNA levels of genes, regulated by miR-21, miR-17 and miR-155 in murine melanoma B16 cells after treatment with μ-oligonucleotides
13. Sequences of RT and PCR primers used in the study
14. The role in regulome, intracellular localization and function of genes, selected for validation of bioinformatic data by qPCR, Western blot or Flow cytometry analysis
15. Comparison of μ-21-ON and μ-21-ON/ μ-17-ON/ μ-155-ON performance in lymphosarcoma RLS_{40} model *ex vivo* and *in vivo*

16. References

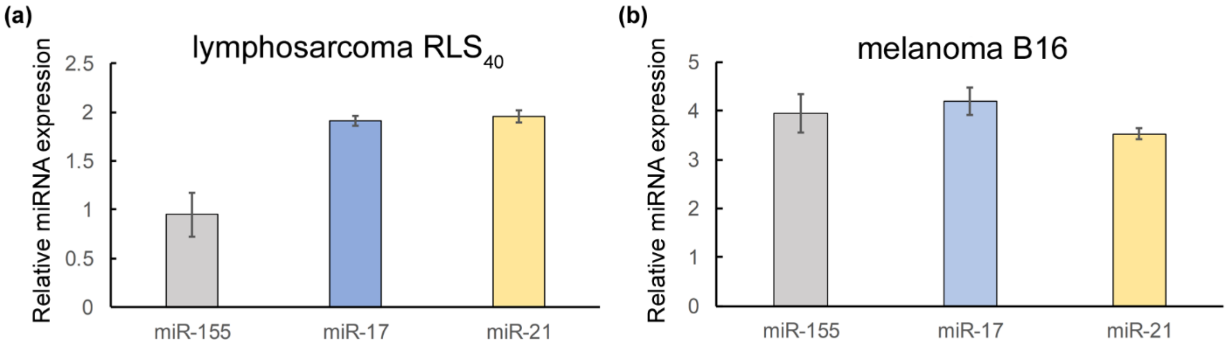

Figure S1. Relative expression of miR-17, miR-21 and miR-155 in lymphosarcoma RLS_{40} (**a**) and melanoma B16 cells (**b**). Expression of miRNAs was measured by stem-loop PCR and normalized to the expression of house-keeping gene *HPRT*.

The initial levels of miR-17, miR-21 and miR-155 were measured in murine melanoma B16 and lymphosarcoma RLS_{40} cells by stem-loop PCR related to mRNA of reference house-keeping gene Hypoxanthine Phosphoribosyltransferase 1 (*HPRT1*). In lymphosarcoma RLS_{40} cells all three miRNAs are expressed in a high level: the level of miR-155 is similar to the level of *HPRT1*, and the expression of miR-17 and miR-21 is twice as high as reference gene (Figure S1a). In melanoma B16 cells miR-155, miR-17 and miR-21 are expressed in a similarly high level, 4-fold above the reference (Figure S1b).

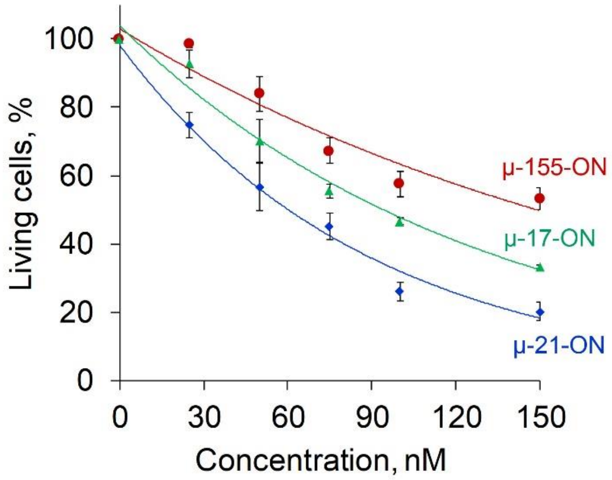

Figure S2. Dose response curves of B16 melanoma cells proliferation depending on concentration of μ-21-ON, μ-17-ON and μ-155-ON. The data was obtained using the xCELLigence cell analysis system 72 h after Lipofectamine™2000-mediated transfection of B16 cells with μ-21-ON, μ-17-ON and μ-155-ON. The data represent mean ± s.e.

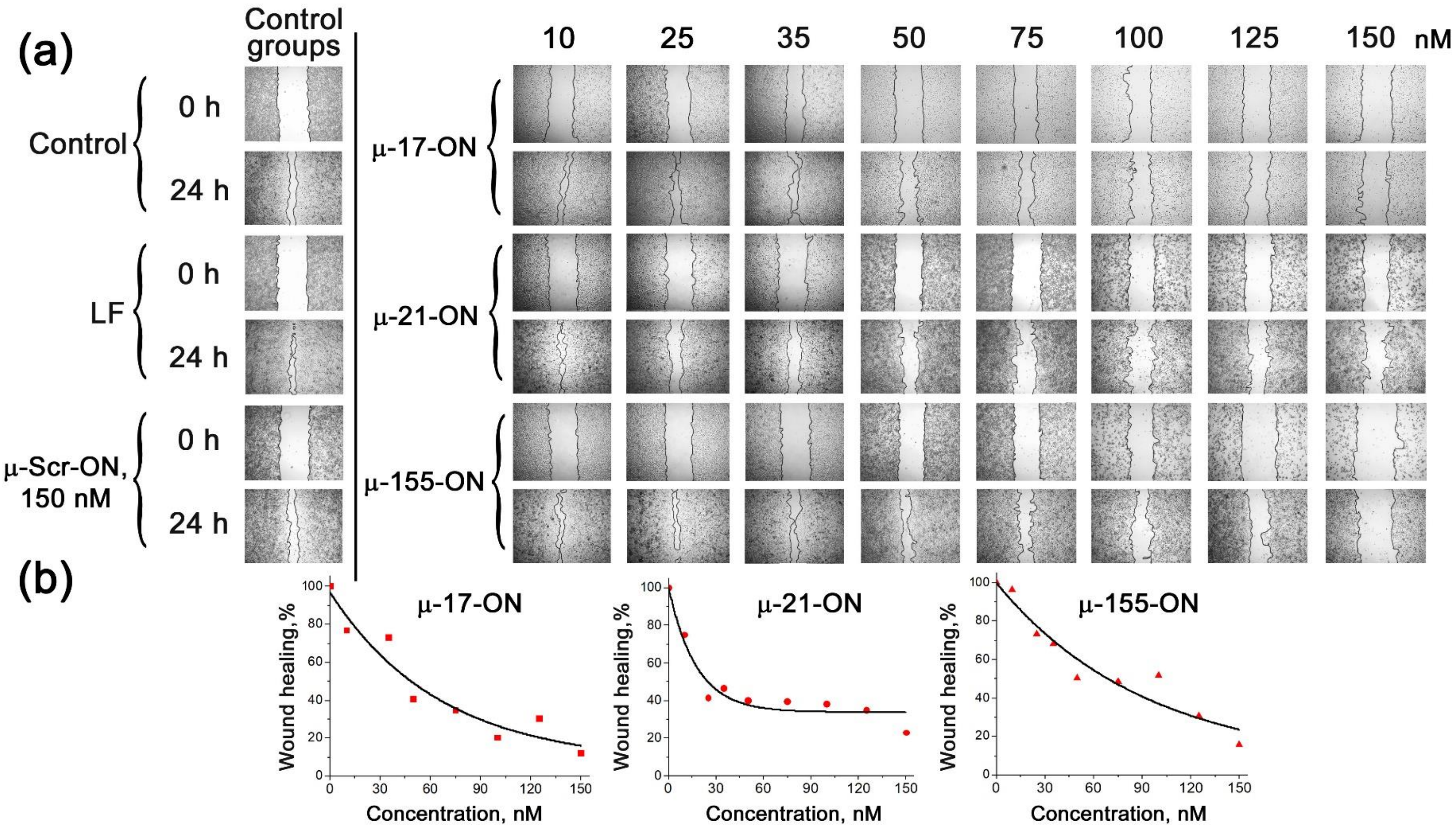

Figure S3. Inhibition of migrative activity of melanoma B16 cells by miRNA-targeted μ-oligonucleotides. (**a**) Photographs of wounds in B16 cell monolayers, at 0 and 24 h after transfection with μ-oligonucleotides. Control – intact B16 cells; LF – cells treated with Lipofectamine™2000 in the absence of oligonucleotides; μ-Scr-ON, μ-17-ON, μ-21-ON and μ-155-ON – cells were transfected with corresponding oligonucleotides as a monotherapy 10 – 150 nM concentration in complex with Lipofectamine™2000. 10-fold zoom. Black lines show wound borderlines; (**b**) Diagrams showing the percent of wound healing depending on the oligonucleotides concentration.

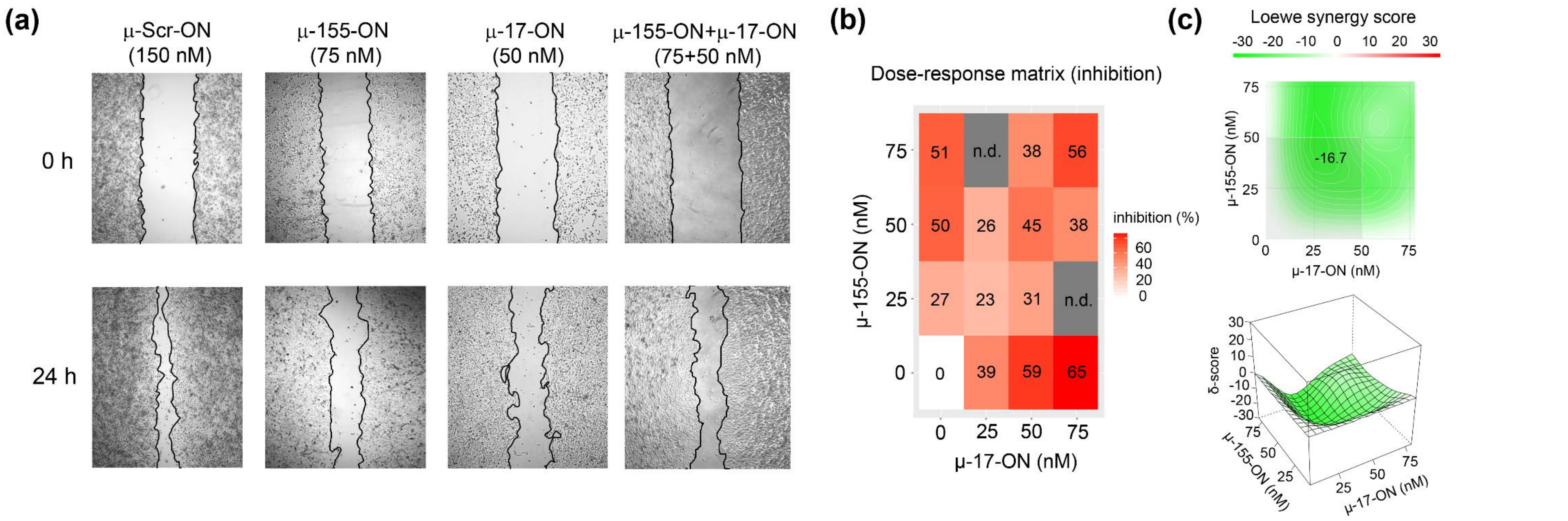

Figure S4. Antimigrative effect of oligonucleotide cocktail µ-17-ON/µ-155-ON on B16 melanoma cells. (**a**) Migration activity of B16 cells, determined by wound healing assay, 24 h after Lipofectamine™2000-mediated transfection with µ-17-ON and µ-155-ON used separately and in combination. µ-Scr-ON – B16 cells treated with control oligonucleotide µ-Scr-ON (150 nM). Concentrations of µ-oligonucleotides are shown on the top; (**b**) Matrix demonstrating inhibition of cell migration by different concentrations of oligonucleotides in combination µ-17-ON/µ-155-ON. n.d. – not determined; (**c**) Loewe score showing synergistic/antagonistic effect of µ-17-ON/µ-155-ON pair on cell migration. The heatmap shows the score values for the areas with the highest synergy and antagonism. The data were analyzed using SynergyFinder web application. The data were analyzed with the SynergyFinder software (https://synergyfinder.fimm.fi/). The synergy score < -10 – antagonism; -10 – 10 –additive effect; > 10 – synergy.

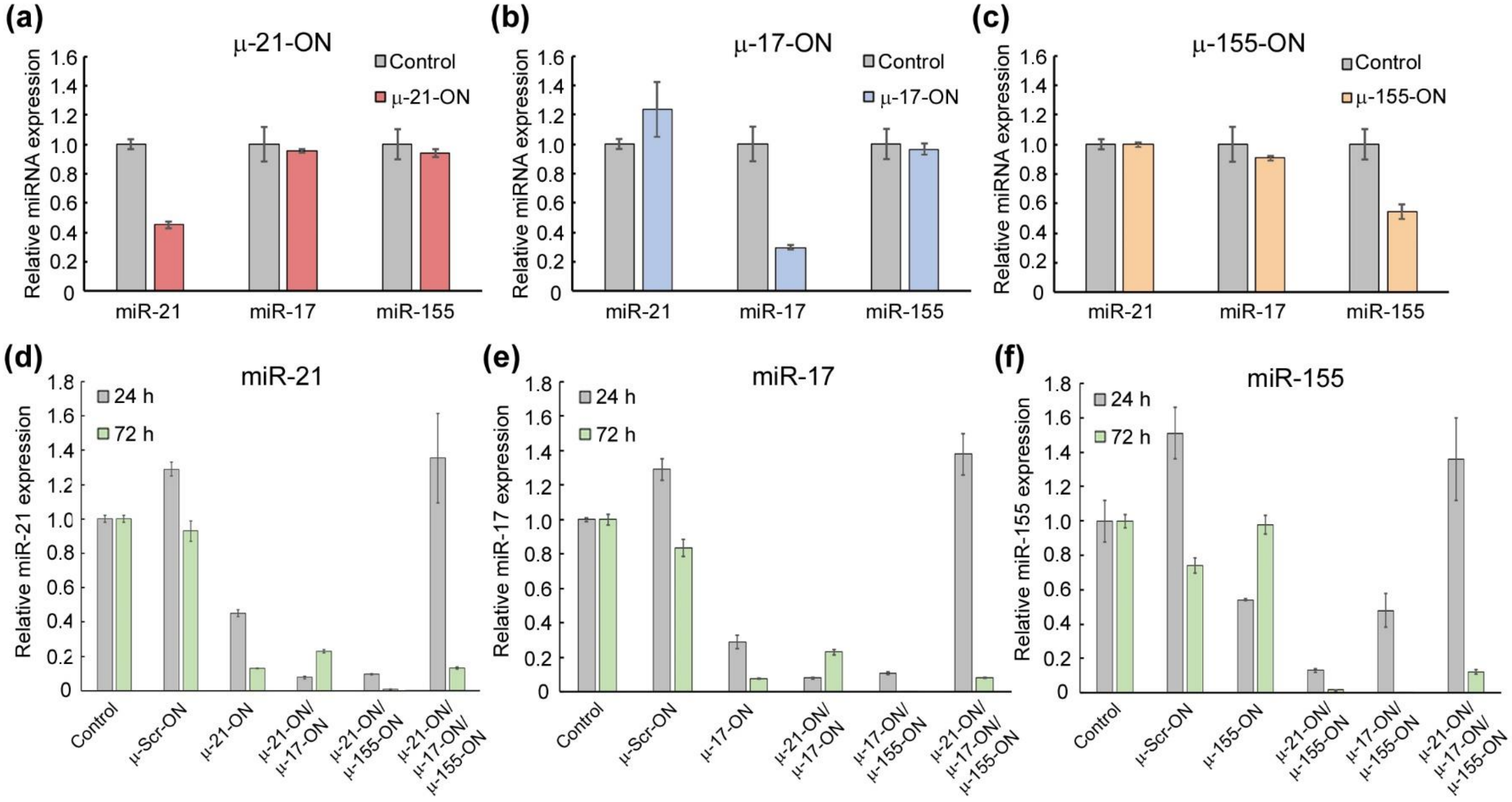

Figure S5. The level of miR-21, miR-17 and miR-155 in melanoma B16 cells after treatment with μ-oligonucleotides separately or in combinations. Relative expression of miRNAs miR-21, miR-17 and miR-155 24 h post-transfection with μ-21-ON (**a**), μ-17-ON (**b**) and μ-155-ON (**c**), respectively. Relative expression of miR-21 (**d**), miR-17 (**e**) and miR-155 (**f**), 24 and 72 h after transfection with μ-oligonucleotides. Control – cells treated with Opti-MEM. μ-Scr-ON, μ-21-ON, μ-17-ON or μ-155-ON – cells treated with single oligonucleotides (150 nM); μ-21-ON/μ-17-ON, μ-21-ON/μ-155-ON, μ-17-ON/ μ-155-ON – cells treated with pairs (75 nM of each ON), μ-21-ON/μ-17-ON/ μ-155-ON – cells treated with triple cocktail (50 nM of each ON). Transfection was performed in complex with Lipofectamine™2000. Expression of miRNAs was measured by stem-loop PCR and normalized to the expression of house-keeping genes *GAPDH* and *HPRT1*.

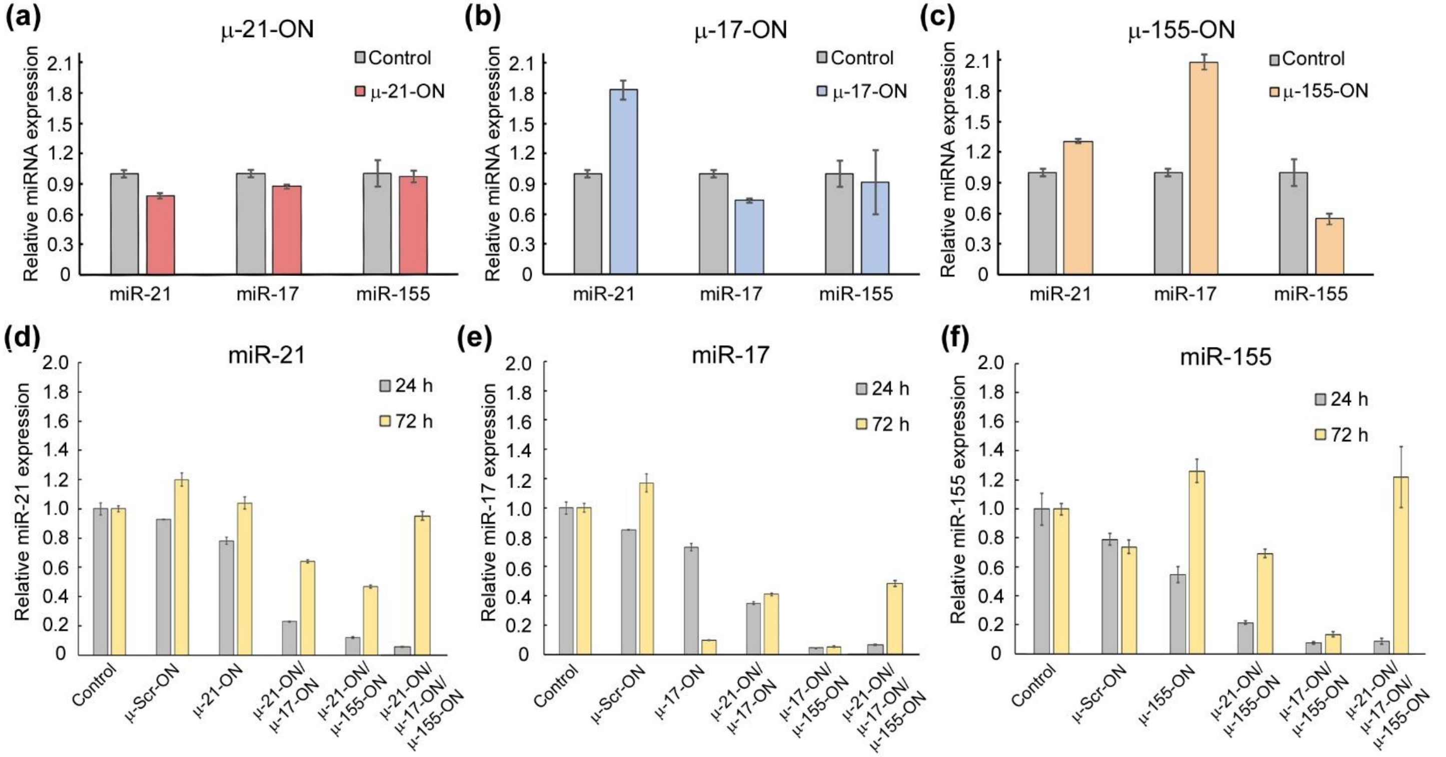

Figure S6. The level of miR-21, miR-17 and miR-155 in lymphosarcoma RLS$_{40}$ cells after treatment with μ-oligonucleotides separately or in combinations. Relative expression of miRNAs miR-21, miR-17 and miR-155 24 h post-transfection with μ-21-ON (**a**), μ-17-ON (**b**) and μ-155-ON (**c**), respectively. Relative expression of miR-21 (**d**), miR-17 (**e**) and miR-155 (**f**), 24 and 72 h after transfection with μ-oligonucleotides. Control – cells treated with Opti-MEM. μ-Scr-ON, μ-21-ON, μ-17-ON or μ-155-ON – cells treated with single oligonucleotides (150 nM); μ-21-ON/μ-17-ON, μ-21-ON/μ-155-ON, μ-17-ON/ μ-155-ON – cells treated with pairs (75 nM of each ON), μ-21-ON/μ-17-ON/ μ-155-ON – cells treated with triple cocktail (50 nM of each ON). Transfection was performed in complex with Lipofectamine™2000. Expression of miRNAs was measured by stem-loop PCR and normalized to the expression of house-keeping genes *GAPDH* and *HPRT1*.

As it can be seen from Figure S5a and Figure S6a, single μ-oligonucleotides (μ-21-ON, μ-17-ON and μ-155-ON) promote efficient and specific downregulation of corresponding miRNAs in the cells. Each oligonucleotide induces specific downregulation of the target miRNA without affecting the levels of other non-complementary miRNAs (Figure S5 a-c, Figure S6 a-c). Paired and triple combinations of

μ-oligonucleotides were also shown to exhibit significant anti-miRNA effect, with the downregulation of miRNAs in melanoma B16 cells being more pronounced and long-lasting in comparison with RLS_{40} model (Figure S5b, Figure S6b).

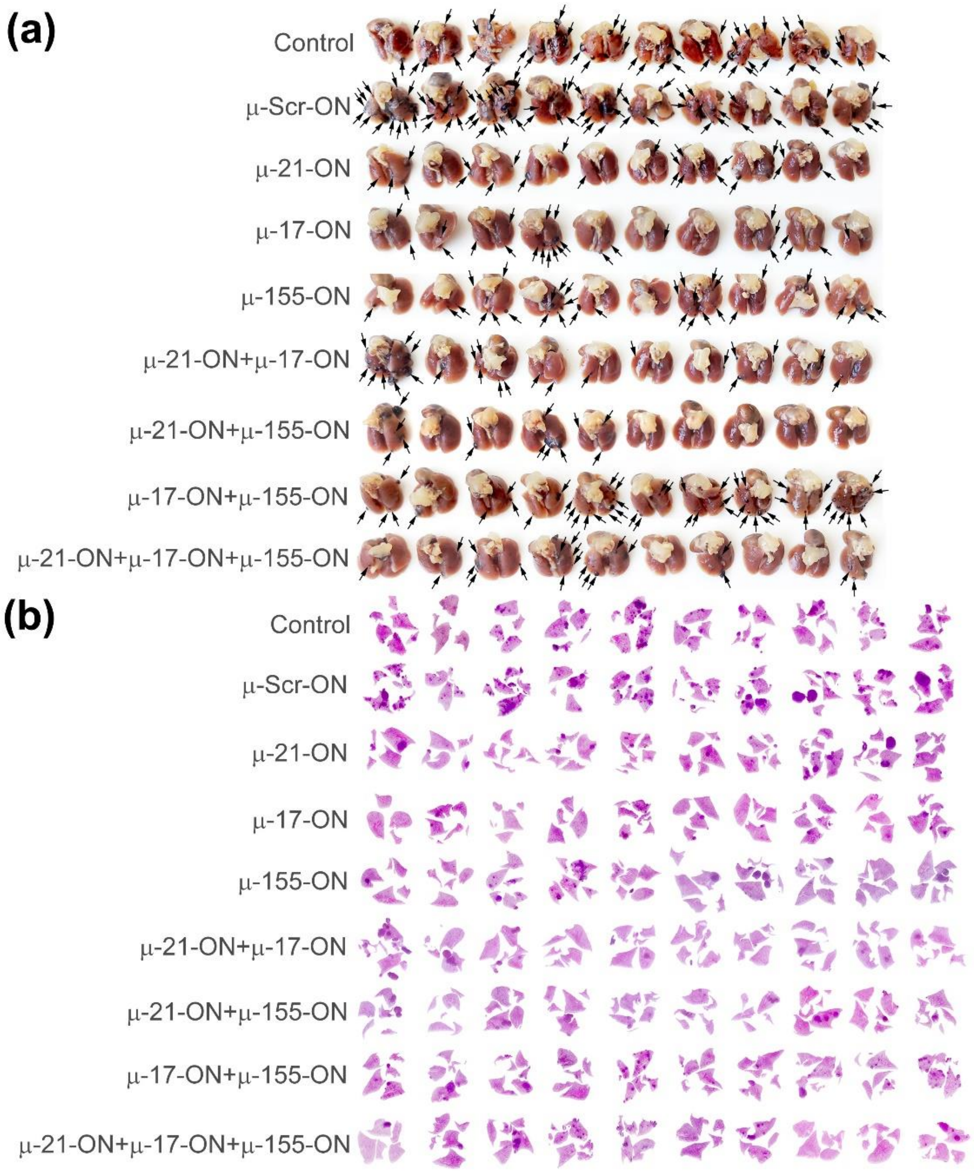

Figure S7. The effect of treatment with miRNA-targeted μ-oligonucleotides on formation of melanoma B16 metastases *ex vivo*. (**a**) Photographs of formalin-fixed lungs with metastases. Arrows indicate examples of metastases.; (**b**) Photographs of lungs histological sections stained with hematoxylin-eosin. Melanoma B16 cells were transfected with μ-oligonucleotides with following intravenous injection to C57Bl/6 mice. Control – cells treated with Opti-MEM, μ-Scr-ON, μ-17-ON, μ-21-ON, and μ-155-ON cells were transfected with corresponding oligonucleotides at 150 nM concentration or with combinations: μ-21-ON/μ-17-ON, μ-21-ON/μ-155-ON, μ-17-ON/μ-155-ON (75 nM each), μ-21-ON/μ-17-ON/μ-155-ON (50 nM each). Transfection was performed with Lipofectamine™2000.

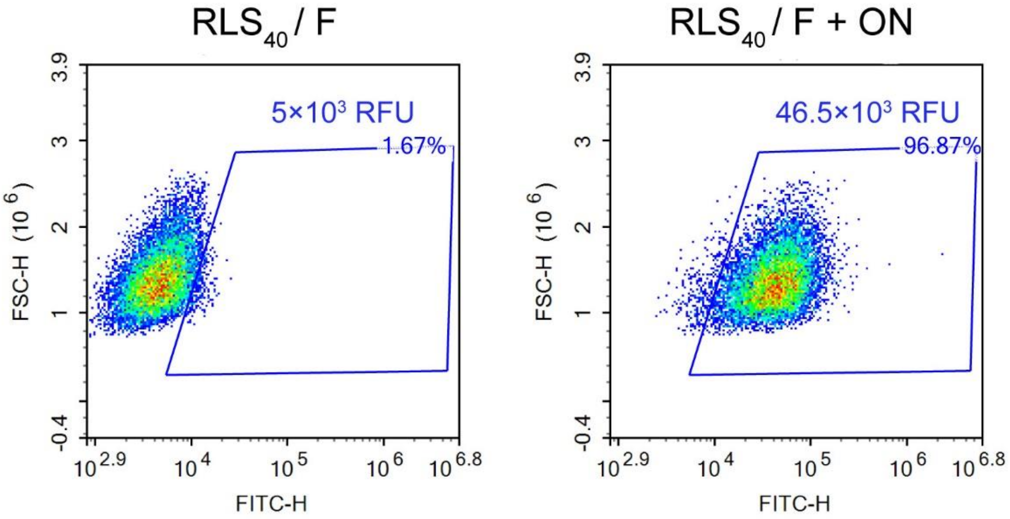

Figure S8. Efficiency of target delivery of FITC-labeled oligonucleotide using folate-equipped liposomes F to lymphosarcoma RLS_{40} cells measured by flow cytometry. The graphs demonstrate the percentage of fluorescent cells and average fluorescence intensity measured in relative fluorescent units (RFU) 4 h after transfection of RLS_{40} cells. Cells were transfected with FITC-labeled oligonucleotide in complex with liposomes F and analyzed using "Novocyte" flow cytometer (ACEA Biosciences Inc, San Diego, CA, USA).

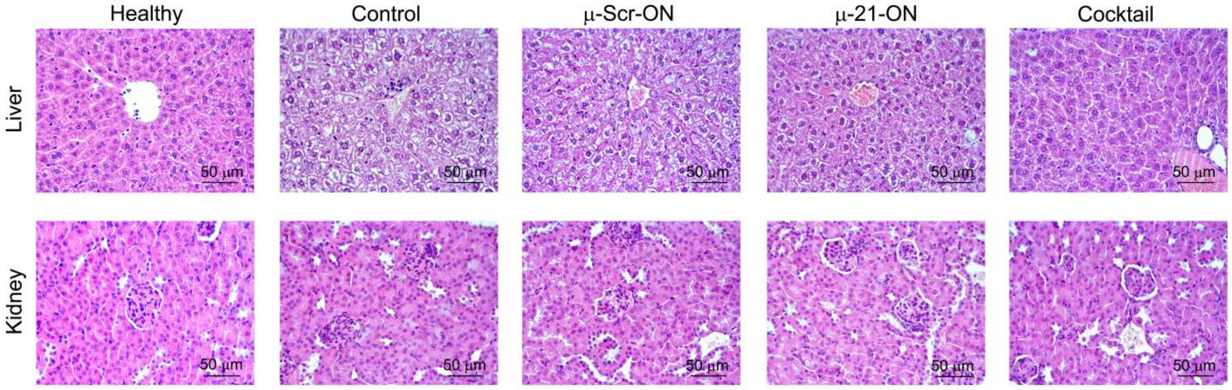

Figure S9. Liver and kidney toxicity of miRNA-targeted treatment with μ-oligonucleotides in CBA mice with lymphosarcoma RLS_{40}. Representative histological images of livers and kidneys of healthy mice and mice with RLS_{40} lymphosarcoma without treatment and after μ-21-ON and μ-21-ON/μ-17-ON/μ-155-ON cocktail administration. Hematoxylin and eosin staining. Original magnification ×400.

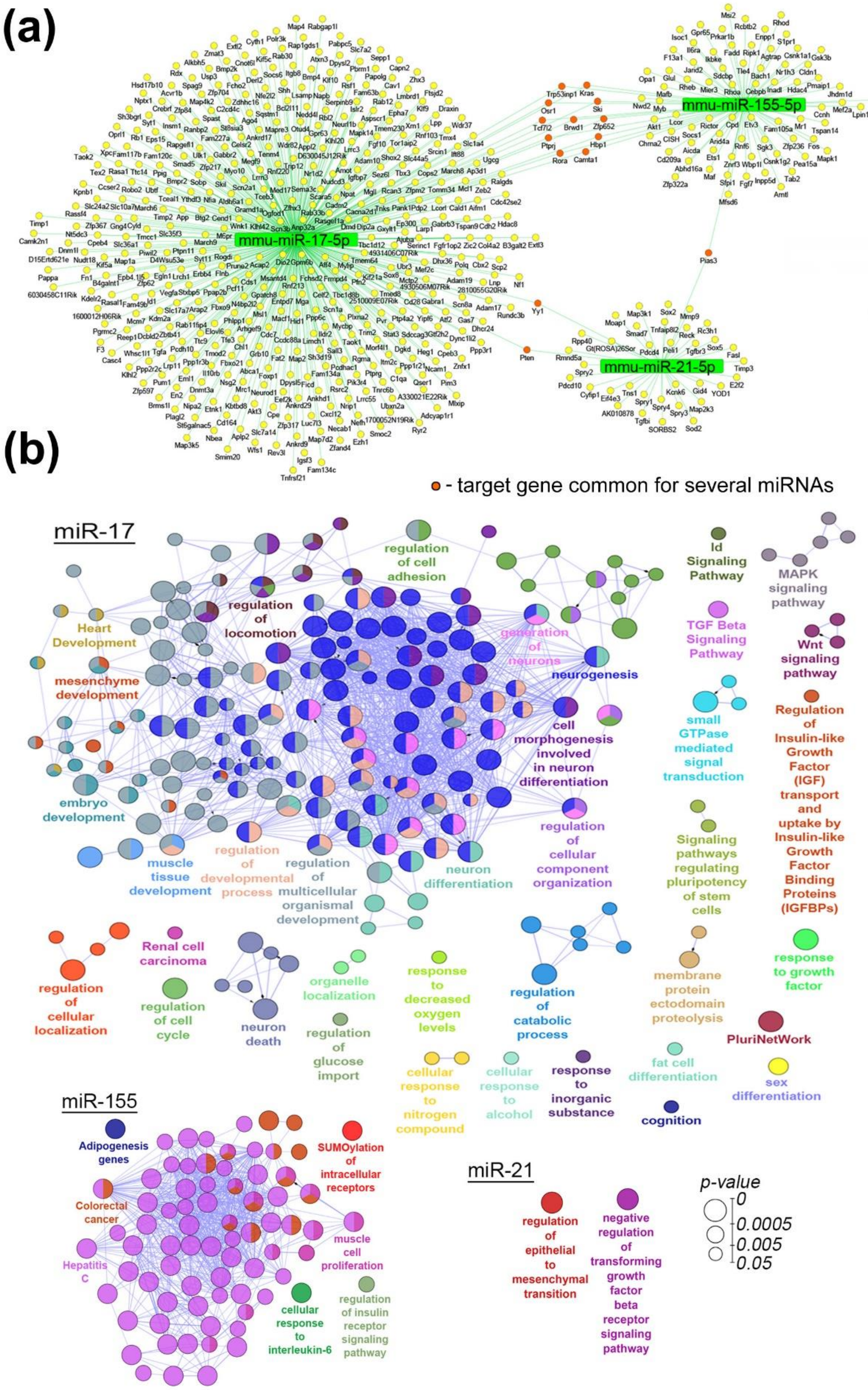

Figure S10. Functional annotation of miRNAs targeted in the study. (**a**) Regulome of miRNAs and their target genes reconstructed using the miRTarBase 8.0 (Mus musculus) database. The analysis was carried out using CyTargetLinker plugin in Cytoscape; (**b**) The network of functional terms enriched with revealed miRNA target genes. Gene set enrichment analysis was performed using ClueGO plugin (Gene Ontology, KEGG, REACTOME, and Wikipathways). Only pathways with $p < 0.05$ after the Bonferroni step down correction were included in the network.

Reconstruction of regulatory network "miRNAs – target genes" using experimentally validated data from miRTarBase database demonstrated its clustered architecture: each analyzed miRNA contained its own unique set of target genes, and only limited number of target genes were found to be common for several miRNAs (Figure S6a). Our analysis showed that miR-17 can modulate expression of 414 target genes, associated with a wide spectrum of biological processes tightly associated with tumor progression, including tumor-related signaling pathways (MAP kinase, Wnt, TGF-β, Id, insulin-like growth factor signaling axes), cell proliferation (regulation of cell cycle, neuron death), cell adhesion and motility (regulation of cell cycle and locomotion, mesenchyme development) (Figure S6b). Interestingly, miR-21 and miR-155 were found to have significantly less number of target genes (37 and 87, respectively) compared with miR-17. Their further functional annotation revealed an ability of miR-21-related regulome to control epithelial-mesenchymal transition (EMT), a key process involved in a regulation of metastasis (Figure S6b), whereas target genes of miR-155 were mainly associated with immune response (major term: hepatitis C, minor terms: measles, yersinia infection, Toll-like receptor, IL-6, IL-5, and IL-17 signaling pathways, etc.) and to a much lesser extent with tumor growth (minor terms: apoptosis, Wnt, ErbB, and VEGF signalings) (Figure S6b).

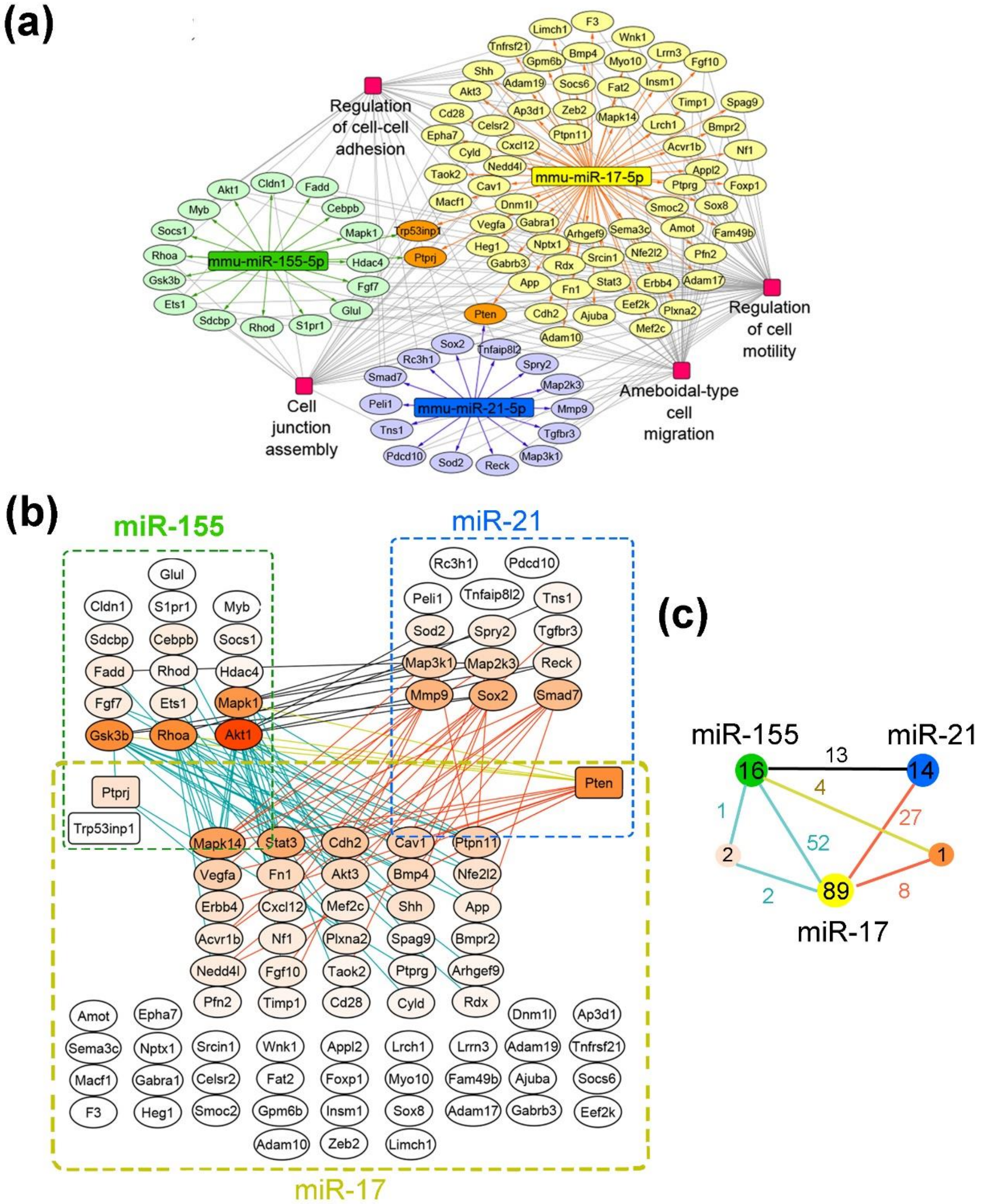

Figure S11. Migration-related targets and possible molecular interactions underlying the effect of miRNA-targeted μ-oligonucleotides on cells motility. (**a**) Regulome of miRNAs and their target genes reconstructed using the miRTarBase 8.0 (Mus musculus) database. The analysis was carried out using CyTargetLinker plugin in Cytoscape; (**b**) Gene-gene interactions between miR-17, miR-21 and miR-155 targets analyzed using String database; (**c**) The general scheme, depicting the number of gene-gene interactions between individual and common target genes of miR-17, miR-21 and miR-155. The numbers on green, dark-blue and yellow circles related to the number of individual target genes regulated by miR-155, miR-21 and miR-17, respectively. The numbers on smaller terracotta and pink circles related to the number of common target genes regulated by miR-21/miR-17 pair and miR-155/miR-21 pair, respectively. Numbers above the junctions related to the interactions between both, individual miRNA targets and between individual and common targets managed by miRNAs. The next interactions are depicted: black – between individual targets of miR-155 and miR-21; terracotta – between individual targets of miR-17 and miR-21 as well as their interconnections with common target of these miRNAs; blue - between individual targets of miR-17 and miR-155 as well as their interconnections with common targets of these miRNAs; yellow – between common target regulated by miR-21/miR-17 and individual targets of miR-155.

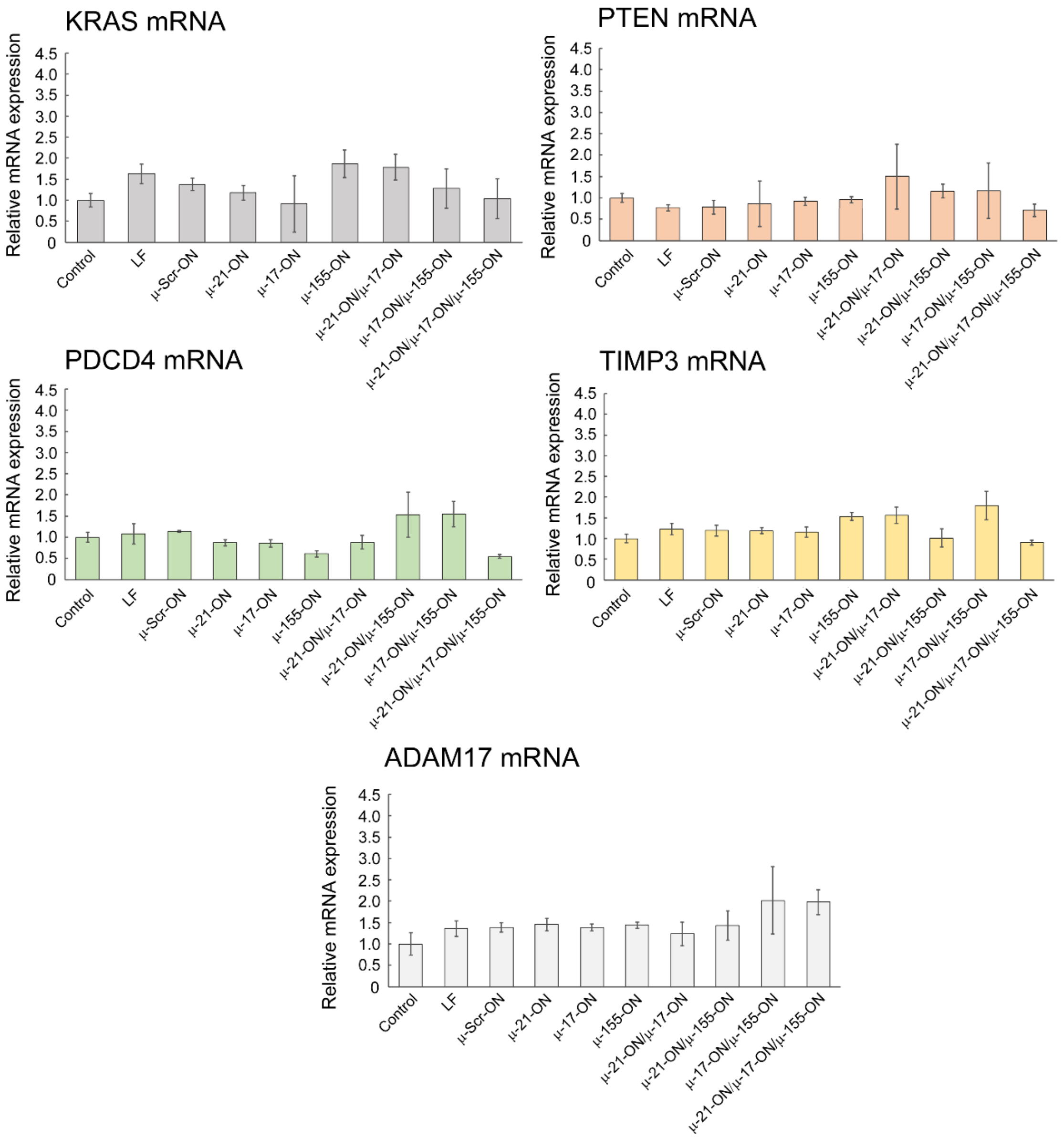

Figure S12. Analysis of mRNA levels of genes, regulated by miR-21, miR-17 and miR-155 in murine melanoma B16 cells after treatment with μ-oligonucleotides. Relative expression of *KRAS*, *PTEN*, *PDCD4*, *TIMP3* and *ADAM17* mRNAs 72 h after transfection of cells with μ-oligonucleotides alone and in combinations, measured by qPCR. Expression of mRNAs was normalized to the expression of housekeeping genes *HPRT1* and *GAPDH*.

Table S1. Sequences of RT and PCR primers used in the study.

Name of the primer	Type of the primer	Sequence (5′-3′)
RT-miR-21	RT	GTCGTATCCAGTGCAGGGTCCGAGGTATTCGCACTGGATAC GACTCAACATCAG
RT-miR-17	RT	GTCGTATCCAGTGCAGGGTCCGAGGTATTCGCACTGGATAC GACCTACCTGCAC
RT-miR-155	RT	GTCGTATCCAGTGCAGGGTCCGAGGTATTCGCACTGGATAC GACACCCCTATCA
miR-21-F	PCR	AGACTAGCTTATCAGACTGA
miR-17-F	PCR	AGACAAAGTGCTTACAGTGC
miR-155-F	PCR	ACTTAATGCTAATTGTGATAGG
Universal Reverse	PCR	GTGCAGGGTCCGAGGT
Hexa-primer	RT	NNNNNN*
Hprt-F	PCR	CATGAAAGCACTCAATAGAAAT
Hprt-R	PCR	TATCTTCCACAATCAAGACATT
Pten-F	PCR	AGGACCAGAGACAAAAAGGGAGTCA
Pten-R	PCR	GCTGGCAGACCACAAACTGAGGA
Pdcd4-F	PCR	CCAGACATTAATCTGGATGTCCCA
Pdcd4-R	PCR	CAGGTTTACGACGGCCTCCA
Mmp9-F	PCR	GTCCAGACCAAGGGTACAGC
Mmp9-R	PCR	GCCTTGGGTCAGGCTTAGAG
Timp3-F	PCR	TCCGGGCCAAAGTGGTGGGA
Timp3-R	PCR	ACGCGCCCTGTCAGCAGGTA
Myc-F	PCR	CCATTGCAGCGGGCAGACACT
Myc-R	PCR	ATCGTCGTGGCTGTCGGGGT
Adam17-F	PCR	CCCATCCGCGACTTGAGAAGC
Adam17-R	PCR	CCGTCCACCACCACGACTCT
Stat3-F	PCR	GCCACGTTGGTGTTTCATAATC
Stat3-R	PCR	TTCGAAGGTTGTGCTGATAGAG
Kras-F	PCR	CTGGGGAGGGCTTTCTTTGTG
Kras-R	PCR	CGATCGTCAACACCTAGTCT
Gapdh-F	PCR	GTGAAGGTCGGAGTCAAC
Gapdh-R	PCR	TGGAATTTGCCATGGGTG

*N – random deoxyribonucleotide

Table S2. The role in regulome, intracellular localization and functions of genes, selected for validation of bioinformatic data by qPCR, Western blot or Flow cytometry analysis.

Gene	miRNA-regulator	Function in regulome: Nodal gene/Individual target and corresponding gene-gene interactions (according to bioinformatic analysis)	Oncogene/ Tumor suppressor	Cellular function (protein)	Cellular localization	Ref
KRAS (Kirsten Rat Sarcoma Viral Proto-Oncogene)	miR-155/miR-17	**Nodal gene** (multiple interactions with miR-155, miR-21 and miR-17 individual targets and other nodal gene managed by miR-21/miR-17 – PTEN)	Oncogene	Stimulation of proliferation	Plasma membrane	[1]
PTEN (Phosphatase and tensin homologue)	miR-21/miR-17	**Nodal gene** (multiple interactions with miR-155, miR-21 and miR-17 individual targets and other nodal gene managed by miR-155/miR-17 – KRAS)	Tumor suppressor	Inhibition of migration, pro-apoptotic factor, modulation of cell cycle	Nucleus, cytoplasm, endoplasmic reticulum (ER), mitochondria-associated membranes (MAMs)	[2,3]
STAT3 (Signal transducer and activator of transcription 3)	miR-17	**Individual target** (multiple interactions with individual targets of miR-155, miR-21 and miR-17 as well as nodal genes – KRAS and PTEN)	Oncogene/ Tumor suppressor	Regulation of apoptosis and proliferation	Cytoplasm, Nucleus	[4–6]
MMP9 (Matrix metalloproteinase 9)	miR-21	**Individual target** (interactions with individual target of miR-155 – Akt1 and targets of miR-17 – RB1, CXCL12, VEGFA, AKT3, MAP3K5, STAT3, MEF2C)	Oncogene/ Tumor suppressor	Regulation of migration and invasion	Cytoplasm	[7,8]
TIMP3 (Tissue inhibitor of metalloproteinase 3)	miR-21	**Individual target** (interactions only with individual targets of miR-17 – NEDD4L and ADAM10)	Oncogene/ Tumor suppressor	Regulation of apoptosis, angiogenesis, invasion, migration and inflammation	Golgi apparatus, vesicles	[9]

PDCD4 (Programmed cell death 4)	miR-21	**Individual target** (interactions only with nodal gene – PTEN)	Tumor suppressor	Inhibition of invasion and migration, pro-apoptotic factor	Cytoplasm, Nucleus	[10]
ADAM17 (A Disintegrin and Metalloprotease 17)	miR-17	**Individual target** (interactions only with miR-17 target TIMP1)	Oncogene	Stimulation of migration and proliferation, pro-inflammatory factor	ER, Cell surface (membrane)	[11–13]

Table S3. Comparison of μ-21-ON and μ-21-ON/μ-17-ON/μ-155-ON performance in lymphosarcoma RLS_{40} model *ex vivo* and *in vivo*.

	**Therapy**	
	**μ-21-ON**	**μ-21-ON/ μ-17-ON/ μ-155-ON**
Tumor volume	3-fold ↓	4-fold ↓
Tumor weight	5-fold ↓	5-fold ↓
Mitotic index (relative to Control/μ-Scr-ON)	18.4-fold ↓ /<br>10.4-fold ↓	**130-fold ↓ /<br>230-fold ↓**
Number of PCNA-positive cells	2-fold ↓	3-fold ↓

References

1. Haidar, M.; Jacquemin, P. Past and Future Strategies to Inhibit Membrane Localization of the KRAS Oncogene. *Int. J. Mol. Sci.* **2021**, *22*, doi:10.3390/IJMS222413193.
2. Bononi, A.; Pinton, P. Study of PTEN Subcellular Localization. *Methods* **2015**, *77–78*, 92, doi:10.1016/J.YMETH.2014.10.002.
3. Song, M.S.; Salmena, L.; Pandolfi, P.P. The Functions and Regulation of the PTEN Tumour Suppressor. *Nat. Rev. Mol. Cell Biol.* **2012**, *13*, 283–296, doi:10.1038/NRM3330.
4. Lee, H.; Jeong, A.J.; Ye, S.K. Highlighted STAT3 as a Potential Drug Target for Cancer Therapy. *BMB Rep.* **2019**, *52*, 415–423, doi:10.5483/BMBREP.2019.52.7.152.
5. Bromberg, J.F.; Wrzeszczynska, M.H.; Devgan, G.; Zhao, Y.; Pestell, R.G.; Albanese, C.; Darnell, J.E. Stat3 as an Oncogene. *Cell* **1999**, *98*, 295–303, doi:10.1016/S0092-8674(00)81959-5.
6. Lu, J.; Xu, S.; Huo, Y.; Sun, D.; Hu, Y.; Wang, J.; Zhang, X.; Wang, P.; Li, Z.; Liang, M.; et al. Sorting Nexin 3 Induces Heart Failure via Promoting Retromer-Dependent Nuclear Trafficking of STAT3. *Cell Death Differ.* **2021**, *28*, 2871–2887, doi:10.1038/S41418-021-00789-W.
7. Tamahashi, U.; Kumagai, J.; Takizawa, T.; Sekine, M.; Eishi, Y. Expression and Intracellular Localization of Matrix Metalloproteinases in Intraductal Papillary Mucinous Neoplasms of the Pancreas. *Virchows Arch.* **2008**, *453*, 79–87, doi:10.1007/S00428-008-0617-6/FIGURES/5.
8. Mondal, S.; Adhikari, N.; Banerjee, S.; Amin, S.A.; Jha, T. Matrix Metalloproteinase-9 (MMP-9) and Its Inhibitors in Cancer: A Minireview. *Eur. J. Med. Chem.* **2020**, *194*, 112260, doi:10.1016/J.EJMECH.2020.112260.
9. Rosewell, K.L.; Li, F.; Puttabyatappa, M.; Akin, J.W.; Brännström, M.; Curry, T.E. Ovarian Expression, Localization, and Function of Tissue Inhibitor of Metalloproteinase 3 (TIMP3) During the Periovulatory Period of the Human Menstrual Cycle. *Biol. Reprod.* **2013**, *89*, 121, doi:10.1095/BIOLREPROD.112.106989.
10. Tran, T.T.; Rane, C.K.; Zito, C.R.; Weiss, S.A.; Jessel, S.; Lucca, L.; Lu, B.Y.; Oria, V.O.; Adeniran, A.; Chiang, V.L.; et al. Clinical Significance of PDCD4 in Melanoma by Subcellular Expression and in Tumor-Associated Immune Cells. *Cancers (Basel).* **2021**, *13*, 1–17, doi:10.3390/CANCERS13051049.
11. Pavlenko, E.; Cabron, A.S.; Arnold, P.; Dobert, J.P.; Rose-John, S.; Zunke, F. Functional Characterization of Colon Cancer-Associated Mutations in ADAM17: Modifications in the Pro-Domain Interfere with Trafficking and Maturation. *Int. J. Mol. Sci.* **2019**, *20*, doi:10.3390/IJMS20092198.
12. Düsterhöft, S.; Lokau, J.; Garbers, C. The Metalloprotease ADAM17 in Inflammation and Cancer. *Pathol. - Res. Pract.* **2019**, *215*, 152410, doi:10.1016/J.PRP.2019.04.002.
13. Saad, M.I.; Rose-John, S.; Jenkins, B.J. ADAM17: An Emerging Therapeutic Target for Lung Cancer. *Cancers (Basel).* **2019**, *11*, doi:10.3390/CANCERS11091218.
